# Software for dataset-wide XAI: From local explanations to global insights with Zennit, CoRelAy, and ViRelAy

**DOI:** 10.1371/journal.pone.0336683

**Published:** 2026-01-02

**Authors:** Christopher J. Anders, David Neumann, Wojciech Samek, Klaus-Robert Müller, Sebastian Lapuschkin

**Affiliations:** 1 Machine Learning Group, Department of Electrical Engineering and Computer Science, Technische Universität Berlin, Berlin, Germany; 2 BIFOLD – Berlin Institute for the Foundations of Learning and Data, Berlin, Germany; 3 Department of Artificial Intelligence, Fraunhofer Heinrich-Hertz-Institut, Berlin, Germany; 4 Machine Learning and Communications Group, Department of Electrical Engineering and Computer Science, Technische Universität Berlin, Berlin, Germany; 5 Department of Artificial Intelligence, Korea University, Seoul, Republic of Korea; 6 Max Planck Institut für Informatik, Saarbrücken, Germany; 7 Centre of eXplainable Artificial Intelligence, Technological University Dublin, Dublin, Ireland; Ariel University, UNITED KINGDOM OF GREAT BRITAIN AND NORTHERN IRELAND

## Abstract

The predictive capabilities of Deep Neural Networks (DNNs) are well-established, yet the underlying mechanisms driving these predictions often remain opaque. The advent of Explainable Artificial Intelligence (XAI) has introduced novel methodologies to explore the reasoning behind complex model predictions of complex models. Among post-hoc attribution methods, Layer-wise Relevance Propagation (LRP) has demonstrated notable adaptability and performance for explaining individual predictions – provided the method is used to its full potential. For deeper dataset-wide and quantitative analyses, however, the manual inspection of individual attribution maps remains unnecessarily labor-intensive and time consuming. While several approaches for dataset-wide XAI-analyses have been proposed, unified and accessible implementations of such tools are still lacking. Furthermore, there is a notable absence of dedicated visualization and analysis software to support stakeholders in interpreting both local and global XAI results effectively. This gap underscores the need for comprehensive software tools that facilitate both granular and holistic understanding of model behavior, as well as easing the adaptability of XAI in applications and the sciences. To address these challenges, we present three software packages designed to facilitate the exploration of model reasoning using attribution approaches and beyond: (1) *Zennit* – a highly customizable and intuitive attribution framework implementing LRP and related methods in PyTorch, (2) *CoRelAy* – a framework to easily and quickly construct quantitative analysis pipelines for dataset-wide analyses of explanations, and (3) *ViRelAy* – an interactive web-application for exploring data, attributions, and analysis results. By providing a standardized implementation for XAI, we aim to promote reproducibility in our field and empower scientists and practitioners to uncover the intricacies of complex model behavior.

## Introduction

While Deep Neural Networks (DNNs) have achieved impressive predictive performance in a wide range of applications (e.g. [1–3]), their complexity is also the cause of a significant limitation: a lack of transparency. Recent advances in XAI (cf. [4–8] for a timely overview), however, allow for a more in-depth investigation of DNN behavior. Here, attribution methods are able to yield local explanations, i.e., attribution scores for all (input) features of individual samples.

Layer-wise Relevance Propagation (LRP) [9,10], with its various purpose-built backpropagation rules [10–12], constitutes a particularly effective approach consistently demonstrating excellent results when utilized according to recommended guidelines [12–15]. Despite its potential, LRP is rarely used to its *fullest capacity*, largely due to a lack of *comprehensive* implementations (cf. Table 1). In particular, a complete implementation of LRP for the popular PyTorch framework, following contemporary recommendations from the literature [7,10,12] is currently lacking. As one of our contributions, we thus aim to make a *versatile* and *flexible* implementation of LRP available to the community, which goes beyond the simple ${\text{LRP}}_{0}$ or ${\text{LRP}}_{\varepsilon }$ variants, often provided as the sole variants of the method [16], despite not being universally recommended [7,12].

**Table 1:** Python frameworks supporting post-hoc attribution for XAI of DNNs. **Framework** lists the name of the framework. **Backend** shows the Python-library the framework is based on. **Propagation Attribution** are the supported propagation-based attribution methods of the framework. **Propagation Rule-map** describes the framework’s support for mapping different rules to layers or parts of a model. **Other Attribution (Notable)** are the *notable* (i.e. non-trivial), non-propagation-based attribution approaches supported by the framework. **Documentation/Tests** highlights the framework’s state of the documentation and tests (with CI).

Framework	Backend	Propagation Attribution	Propagation Rule-map	Other Attribution (Notable)	Documentation Tests
Zennit (ours)	PyTorch	Common LRP [10] Uncommon/Custom LRP Guided Backprop [37] Excitation Backprop [45]	Built-In Custom Canonization	SmoothGrad [42] Integrated Gradients [38] Occlusion [34]	Full Usage API Tutorials Fully Tested + CI
Captum [16]	PyTorch	${\text{LRP}}_{\varepsilon }$ [10] DeepLIFT(+Shap) [44,48] Guided Backprop [37]	None	SmoothGrad [42] Integrated Gradients [38] Conductance [39,40] GradientShap [48] KernelShap [48] Grad-CAM [41] Occlusion [34] LIME [47] Shapley Values [91,92]	Full Usage API Tutorials Fully Tested + CI
TorchRay [36] (unmaintained)	PyTorch	Guided Backprop [37] Excitation Backprop [45]	None	Grad-CAM [41] Occlusion [34] LIME [47] RISE [35] Extremal Perturbation [36]	Joint Usage + API Examples Benchmarks
iNNvestigate [57]	TensorFlow/ Keras	Common LRP [10] PatternAttribution [46] DeepLIFT [44] Guided Backprop [37]	Built-In	SmoothGrad [42] Integrated Gradients [38]	Usage in Readme API Tutorials Fully Tested + CI
DeepExplain [60] (unmaintained)	TensorFlow/ Keras	${\text{LRP}}_{\varepsilon }$ [10] DeepLIFT [44]	None	Integrated Gradients [38] Occlusion [34] Shapley Values [91,92]	Usage in Readme Examples Tests + CI

If employed correctly, local XAI has the potential to point out previously unknown but interesting model behavior, or biased and artifactual predictions [17,18]. With very large datasets, however, a thorough (manual) analysis of attribution results—e.g. for the understanding and verification of model behavior, or the discovery of systematic misbehavior—is very labor- and time-intensive. Still, further insight into a model’s inner workings, beyond local attributions, is crucially necessary, e.g. to understand global model behavior or to uncover hidden Clever Hans (CH) [19,20] traits. Recent innovations, such as Spectral Relevance Analysis (SpRAy) [20,21] or Prototypical Concept-based Explanations (PCX) [22], have streamlined the process of analyzing models by automating significant aspects of the analysis workflow. When combined with informative visualizations, these approaches facilitate the identification and understanding of prediction strategies employed by DNNs.

In this paper, we introduce three software packages targeted at scientists and practitioners to explore the reasoning of Machine Learning (ML) models based on dataset-wide XAI:

1. With *Zennit* (source: https://github.com/chr5tphr/zennit, documentation: https://zennit.rtfd.io) we provide a highly customizable, yet intuitive local XAI framework for PyTorch, centered around rule-based approaches such as LRP. Leveraging the Module architecture of PyTorch, *Zennit* enables an easy yet flexible implementation and application of such rule-based techniques and provides the user with a comprehensive set of built-in attribution methods.
2. *CoRelAy* (source: https://github.com/virelay/corelay, documentation: https://corelay.rtfd.io) further processes attribution information (and potentially other data sources) and can be employed to rapidly construct complex, comprehensive analysis workflows that span entire datasets. Examples of such pipelines include SpRAy or PCX, which may involve pre-processing, embedding, and clustering steps. The framework seeks to optimize analysis efficiency by re-using (partial) pipeline results whenever possible. By utilizing cached results within and across pipeline executions, it minimizes the need for redundant computations, such as those triggered by changes to parameters, thus reducing overall processing time.
3. *ViRelAy* (source: https://github.com/virelay/virelay, documentation: https://virelay.rtfd.io) offers a user-friendly interface to explore analysis results, produced by *Zennit* and *CoRelAy*, through an interactive web application. As users delve into the exploration of attributions, clusterings, and embeddings, they can easily import, export, bookmark, and share specific findings with their peers, facilitating collaboration and knowledge sharing.

**Fig 1:**
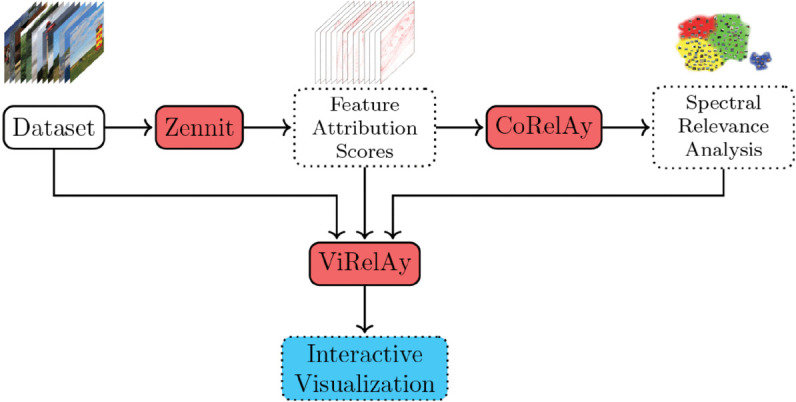
Relation between our software frameworks *Zennit, CoRelAy*, and *ViRelAy.* Given a dataset, *Zennit* produces feature attribution scores (e.g., using LRP), which are used by *CoRelAy* to conduct, e.g., a SpRAy. *ViRelAy* uses the dataset and outputs of both other frameworks to present an interactive visualization.

Together, *Zennit*, *CoRelAy*, and *ViRelAy* empower users to conduct comprehensive and insightful explorations of complex models and large-scale datasets through XAI. See Fig 1 for an illustration of how our proposed software frameworks interconnect. The integrated and flexible approach they offer enables users to move beyond passively observant XAI, allowing for informed interventions driven by meaningful insights. For instance, our software packages have enabled researchers to identify systematic biases in DNN models [21] trained on ImageNet [23], demonstrating the potential of these tools.

Each software package is thoroughly documented and equipped with a comprehensive test suite and static code analysis tools that continuously monitor the software’s functionality and source code quality through a Continuous Integration (CI) pipeline (cf. Section Testing and quality assurance).

Since their inception, the projects have seen significant growth, with an expanding community of contributors, pull requests, new releases, and adoption in other software. Various research works did utilize our frameworks, including works on feature attribution for regression problems [24], debugging and improving Neural Networks (NNs) [21,25], preventing catastrophic forgetting through relevance-based neural freezing [26], concept-based attribution [27,28], improving Model Parameter Randomization Tests (MPRTs) [29], model quantization [30], applications in histopathology [31], clinical gait analysis [32], and even for introducing a novel relevance-based alternative to gradient descent [33]. We invite the community to join our efforts in making XAI more accessible and user-friendly.

## Related work

Our three software packages provide implementations for three different stages of model understanding. In this section, we first introduce the underlying methods which are implemented by our frameworks, and then introduce related software frameworks for each stage: feature attribution, analysis implementation, and visualization. For a detailed comparison of alternative frameworks, see Section Comparison to alternative frameworks.

### Explainable Artificial Intelligence (XAI)

Many kinds of ML models, in particular NNs, are considered black-boxes, i.e. although their theoretical underpinnings and the mechanics of their functioning are well-understood, these systems have become so complex that understanding them fully or even only in part, has grown increasingly intractable. The field of XAI comprises a set of theories and methods that aim to provide scientists and practitioners with transparency regarding the inner-workings of ML models. In particular, it aims at providing explanations for the decision-making processes of discriminative models, with the intention of validating that the learned prediction strategies are sound or identifying spurious behavior. The field can broadly be split into two branches: *local* and *global* XAI. While local XAI concentrates on providing explanations for the predictions of a model for individual samples, global XAI focuses on gaining a comprehensive understanding of the model itself. As our software frameworks mainly concern local XAI, we put our focus on this particular branch.

#### Local XAI and feature attribution.

Methods for local XAI provide explanations for individual predictions of a ML model. Most commonly, this is done by attributing an importance score to each feature towards the models’ decision in input or latent space, consequently called *feature attribution*. In most cases, the importance of the input features is visualized as a heatmap aligned to the input space.

Without claim of completeness, feature attribution methods can be broadly categorized into the following four categories: (1) *Perturbation analysis* [34–36], in which input samples are explicitly perturbed with the goal of identifying the extent to which input variables locally affect the model’s output. (2) *Sensitivity analysis* [37–42], which analyzes local first-order approximations of the model’s output with respect to input perturbation via the gradient or variations thereof, indicating the model’s local sensitivity to this change (3) *Decomposition-based methods* [9,10,43–46], which propagate sum-decompositions of a model’s output on a per-layer basis down until the input features, and (4) *surrogate model-based methods* [47–49], which create inherently interpretable surrogate functions to estimate the target model’s behavior in a local neighborhood.

See Appendix Feature attribution approaches for a high-level description of some common feature attribution approaches.

#### Layer-wise relevance propagation.

**Fig 2:**
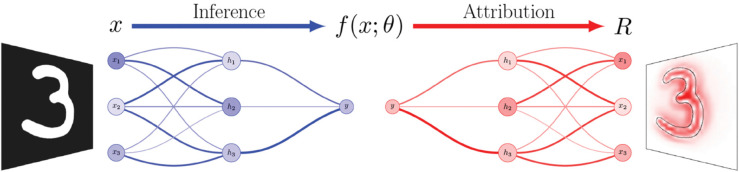
An overview of how LRP operates. First inference is performed on the input sample *x* by performing a forward pass through the model f(x;θ), resulting in a prediction *y*. The prediction score is then propagated backwards through the layers of the model until the input layer is reached. The resulting attribution *R* reaches all latent and input components of the model and can then be visualized as a heatmap.

Among the various feature attribution approaches, this paper is chiefly concerned with LRP, as it plays a key part in the primary use case covered by our software packages. LRP [9,10,43] is a decomposition-based feature attribution method for NNs or NN-like ML models. It operates by backpropagating a model’s prediction from the output to the input, using specialized propagation rules. The propagation process adheres to a conservation principle: the relevance a neuron receives must be redistributed to the neurons it is connected to in the previous layer in equal measure. This means that the sum of the relevances attributed to the input neurons is equal to the value of the output neuron that is being explained. An overview of the general approach is illustrated in Fig 2. For details on LRP and its different rules, see Appendix Layer-wise relevance propagation: Details. The basic principles of LRP can be recovered in various other feature attribution approaches, allowing for a unified understanding of their underlying mechanisms. Subsequently, these methods can also be implemented using the LRP framework, demonstrating its versatility and potential as a generalized framework for explainability. This includes Deep Taylor Decomposition (DTD) [43], Excitation Backprop [45], Gradient × Input [44], CAM [50], Grad-CAM [41], and DeepLift [44].

#### Spectral relevance analysis.

**Fig 3:**
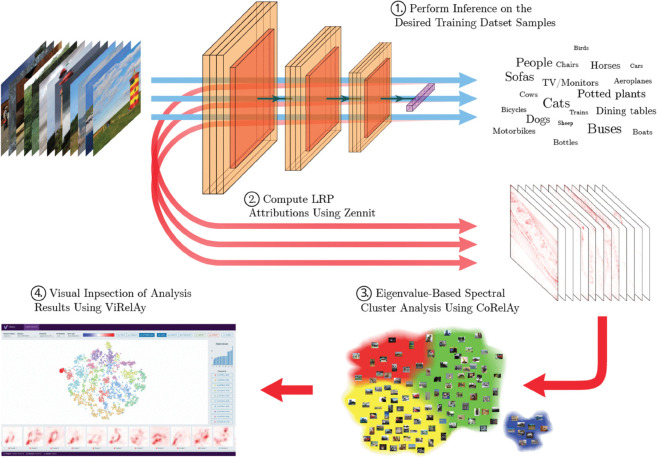
The workflow of SpRAy as implemented within our software frameworks. (1) First, the model is used to perform inference on all samples of a dataset. (2) The classification decisions are then explained using a suitable LRP variant with the help of *Zennit*, resulting in attributions and heatmaps for all samples and classes of interest. (3) Then, an eigenvalue-based spectral cluster analysis is performed using *CoRelAy*, in order to identify different prediction strategies within the analyzed attribution data. (4) The resulting embeddings (e.g. the raw spectral embedding or e.g. a t-SNE or UMAP embedding based thereon) and clusterings (e.g. k-Means) can then be visualized in *ViRelAy* to identify possible characteristic prediction strategies or CH behavior of the model. This information can be used to improve the model or the dataset.

Feature attribution for individual input samples does not provide the means to understand the systematic behavior of a model. Although global XAI methods may provide us with a broad understanding of a model’s inner workings, they usually cannot be used to identify individual defects in the decision process of the model. SpRAy [20,21] solves this issue by analyzing patterns of local feature attributions over the whole data set to provide a global understanding of the model behavior. A rough outline of the method is shown in Fig 3. For more details on the specific pipeline, see Appendix Spectral relevance analysis: Details. For comprehensive overview over techniques that apply (the insights derived from) XAI methods to improve ML models, please refer to [25].

### Attribution frameworks

Prior to this work, various software frameworks have been developed to compute feature attributions using different deep learning libraries. A notable early example of a comprehensive XAI framework is the *LRP Toolbox* [51], which provides implementations for various recommended LRP decomposition rules for the Caffe Deep Learning Framework [52], as well as for Matlab [53] and Python [54] (using NumPy [55] and CuPy [56]) through custom NN interfaces. Similar to the Caffe framework, which has reached the end of its life cycle in 2018, the LRP Toolbox is no longer actively maintained.

The *iNNvestigate* [57] framework, built on TensorFlow [58] and Keras [59], offers implementations for LRP and other attribution methods. While it simplifies applying numerous approaches to existing Keras models, its architecture makes customization challenging. Implementing custom backpropagation rules and rule compositions requires significant effort, limiting the framework’s immediate applicability to novel architectures.

*DeepExplain* [60] is another attribution framework for TensorFlow and Keras, which supports a number of gradient and perturbation-based attribution methods. ${\text{LRP}}_{\varepsilon }$ and DeepLIFT are the only decomposition-based attribution methods supported and the architecture of the framework does not allow for the easy implementation of other rule-based attribution methods, as it is lacking the infrastructure for applying separate rules to individual layers. At the time of our research, DeepExplain had not received maintenance in several years, suggesting that is no longer actively maintained, and thus no longer constituting a viable option.

*Captum* [16], which is tightly integrated into PyTorch, provides a broad spectrum of attribution methods. Although it features high customizability, it lacks support for layer-type-specific implementations of decomposition rules required for LRP. It requires a substantial amount of work to employ state-of-the-art recommendations for LRP.

*TorchRay* [36], another PyTorch-based framework, offers a broad spectrum of attribution methods but does not provide support for LRP.

*OpenXAI* [61] and *Quantus* [62] take a different approach by providing tools to evaluate XAI methods, thus focusing on reproducibility in XAI research.

### Pipelining frameworks

Even though we are proposing a framework that was specifically designed for SpRAy and SpRAy-like data processing pipelines for which no alternatives exist, there are many general-purpose software packages available that can facilitate the construction of such pipelines, albeit with a significant amount of additional work, as many of the SpRAy-related functionality has to be re-implemented.

In addition to our own framework, *CoRelAy*, other notable frameworks include Scikit-Learn [63], Luigi [64], and Apache AirFlow [65]. Although *Scikit-Learn* is primarily a machine learning framework, it also provides native pipelining functionality. Similar to *CoRelAy*, these functionalities are optimized for single-machine workflows, while *Luigi* is designed specifically for long-running batch jobs with a client-server model. *Apache AirFlow* offers even more advanced features for distributed and high-performance computing. In contrast, *CoRelAy* was developed as a single-machine tool for XAI to allow rapid composition of analysis pipelines. Caching of the results allows for quick adaptation when parts of the pipeline are changed. Most notably, its interface between *Zennit* and *ViRelAy* allows to swiftly see changes of the model in *ViRelAy*’s graphical interface, ranging from changes seen in feature attributions to changes of the pipeline.

### XAI visualization applications

While no ready-made software with the visualization and exploration capabilities of *ViRelAy* exists, there are other tools for visualizing the results of XAI methods.

The Captum framework, for example, offers its own built-in visualization tool called *Captum Insights* [16]. While it is most comparable to *ViRelAy*, as it allows users to view attribution maps together with their respective input samples and classification results, there are, besides basic data filtering capabilties, no further tools for exploring the results of XAI outputs or for identifying interesting model behavior.

The *interpretML* [66] software package is a blend of a framework for training and explaining glass-box – i.e. inherently interpretable – ML models, and a dashboard for visualizing samples, feature importance scores and the performance of the model.

Finally, *explainerdashboard* [67] offers a Scikit-Learn-compatible interface for visualizing feature importance, dependence, and interactions of models. It also provides visualizations for model performance, enabling users to investigate individual predictions for specific samples. The dashboard offers an extensive feature set for understanding simple machine learning models, yet it is limited to tabular data and does not support the visualization of image-based feature analyses.

## Attribution with Zennit

*Zennit* is a feature attribution framework for PyTorch [68] with a primary focus on the rule-based approach of LRP [9]. It aims to offer a simple and intuitive user experience, while still remaining flexible and easy-to-modify. This enables *Zennit* to optimally align the method to the characteristics of the analyzed model (or parts thereof) [7,10,12], which is highly important for obtaining good results. This is were other frameworks are unsuccessful, as their implementations of LRP are rigid, and limit the user’s capabilities to mapping propagation rules to parts of the model architecture preventing the optimal adaptation of LRP to novel NN architectures.

*Zennit* leverages three core components of PyTorch: (1) the Module class, (2) its automatic differentiation engine, and (3) its infrastructure for hooking into life cycle events of modules. The Module class forms the basis for NN layers, enabling *Zennit* to identify the model’s architecture. LRP is a method based on the propagation of the model’s output back through the NN to attribute relevance to individual neurons. In NNs, this process is reminiscent of the backpropagation of gradients, which is implemented in PyTorch via its automatic differentiation engine “autograd”. *Zennit* appropriates the autograd system for the implementation of its own (modified) backpropagation algorithm. This is accomplished by utilizing the hook infrastructure of PyTorch, which allows *Zennit* to attach callback functions to the life cycle events of tensors, computation graph nodes, and modules. Two such life cycle events are the forward and the backward pass through a module, which can be hooked into to modify or replace the output or the gradient respectively. The backward hook is exploited by *Zennit* to apply the decomposition rules of LRP and other methods, and to replace the gradient with the propagated relevance scores. Backward hooks, as well as pre-forward and forward hooks are also used to set up the required infrastructure. Finally, *Zennit* also provides implementations for more simple black-box attribution methods, such as SmoothGrad [42] and Integrated Gradients [38]. These methods do not require a layer-wise attribution system and are instead straight-forward functions of the gradient of the model. Additionally, they can be arbitrarily combined with rule-based approaches to obtain more complex feature attribution approaches (e.g., a combination of SmoothGrad and LRP).

### Rule-based attributions

**Fig 4:**
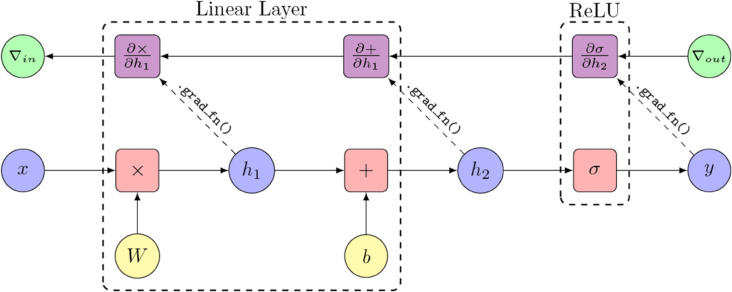
A schematic representation of a PyTorch computation graph and its corresponding gradient computation graph. An example for a linear layer followed by a ReLU activation function is shown.

Rule-based attribution methods assign different propagation rules to individual components within a model, depending on their functions and context. In PyTorch, when an operation is performed, e.g. two tensors are added together, a computation graph is built implicitly in the background. If automatic differentiation is enabled for the tensors, i.e. the requires_grad property is set to True, then PyTorch also builds a gradient computation graph, by adding a grad_fn to the tensor, which contains the function required to compute the gradient of the operation that resulted in the tensor. Multiple such operations can be grouped together in a layer that is implemented as a sub-class of PyTorch’s Module class. An example of such a computation graph is the sequence of a linear layer module followed by a Rectified Linear Unit (ReLU) non-linearity module. An illustration thereof together with its corresponding gradient computation graph is shown in Fig 4.

Modules provide *hooks* to which *callbacks* can be registered that are invoked at certain points in a module’s life cycle. There are multiple *hook* variants, e.g. *pre-forward hooks* are invoked before the forward pass through the layer is performed, *forward hooks* are invoked after the forward pass through the module, and *backward hooks* are invoked after the gradient of the module has been computed.

**Fig 5:**
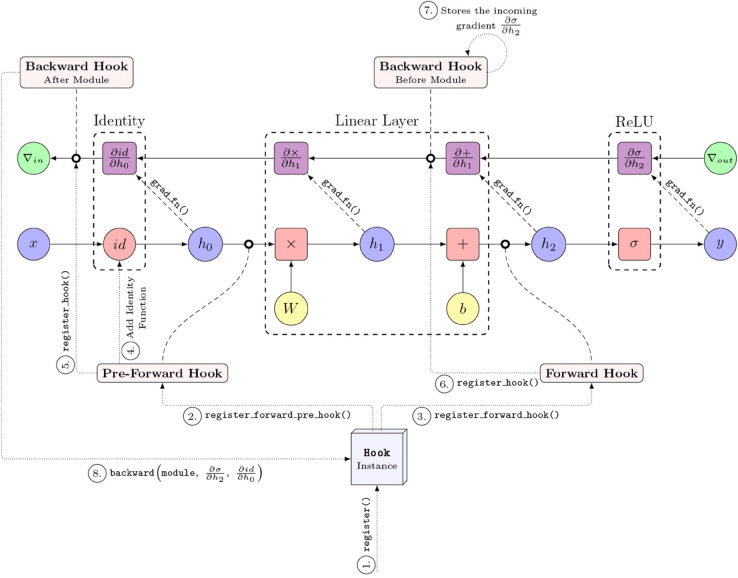
A computation graph modified by *Zennit.* The gradient computation graph of PyTorch is utilized to implement decomposition-based methods such as LRP.

To implement a backpropagation rule for a layer, *Zennit* first attaches *pre-forward* and a *forward hook* to the module. In the pre-forward hook, *Zennit* injects an additional identity function node into the computation graph right before the module, to which a *backward hook* is attached. In the forward hook, a *backward hook* is registered with the module output, which, once invoked, stores the incoming gradient from the upstream layer, in order to provide access during the injection of the backward hook. When the backward hook of the identity function is executed, it accesses the gradient stored by the backward hook of the module output, applies the backpropagation rule to compute the desired attribution scores, and overwrites the gradient passed to the downstream layer with the redistributed attribution scores. This process is depicted in Fig 5, showing the same computation graph as in Fig 4, as modified by *Zennit*.

This functionality is implemented in *Zennit*’s custom Hook class. Rules for attribution methods are implemented by sub-classing the Hook class and implementing the backward method, which is invoked by the backward hook of the injected identity function. This provides a flexible and intuitive interface to trivialize the implementation of additional rules. Specifically for LRP-based rules, *Zennit* offers a streamlined approach with the BasicHook class, enabling the definition of LRP rules based on rule components which require the modification of a layer’s parameters, inputs and value accumulation behavior during the forward and backward passes. All popular rules for LRP (for an overview see [10]), as well as rules for other approaches, such as GuidedBackprop [37] and ExcitationBackprop [45], are provided as part of the framework.

### Mapping rules with composites

**Fig 6:**
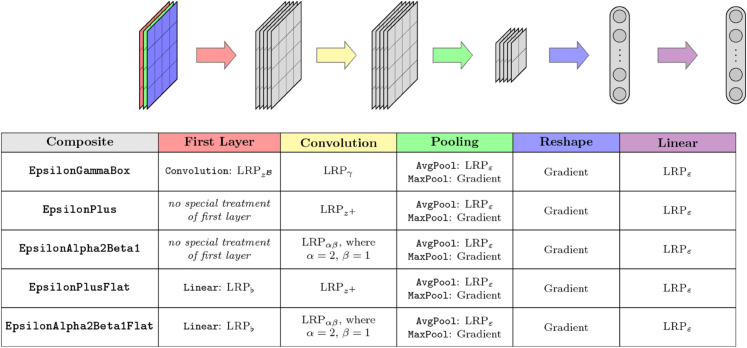
A list of pre-made rule composites provided by *Zennit.*

A critical component for a successful implementation of rule-based attribution methods is an efficient approach to assign a set of propagation rules to specific layers and model components. *Zennit* implements this component through *composites*, which are defined as mappings from Module-properties to rules. Examples for common Module-properties are the name of a layer or its type of function, (hyper-)parameters, or global position within the model’s architecture. Basic composites are defined through a module_map function. This function is expected to return a *template-rule* given the provided Module-properties. This *template-rule* will be copied and registered for each matching layer. To compute an attribution, composites temporarily register their rules to all matching layers and thus modify the gradient of the full model to instead represent the attribution scores. Although the module_map structure – mapping individual Modules to backpropagation rules – provides the most freedom, *Zennit* provides subclasses of the Composite base class, which constitute configurations of assignments from Module type, -property or -position to decomposition rules. One such example is the SpecialFirstLayerMapComposite, which assigns rules based on layer types except for the first linear layer, which receives a special rule. This format is the basis for the in literature recommended LRP-based composites [7,10,12] for feed-forward NNs, which *Zennit* provides for user convenience. One such recommendation is the EpsilonGammaBox composite, which assigns the ${\text{LRP}}_{\varepsilon }$-rule to dense layers, the ${\text{LRP}}_{\gamma }$-rule to convolutional layers, and the $z^{ℬ}$-rule (or box-rule) to the convolutional layer at the input [10]. Fig 6 provides a list of available composites and the rule assignments they constitute. See Appendix Layer-wise relevance propagation: Details for a detailed definition of backpropagation rules.

### Temporary model modification with canonizers

**Fig 7:**
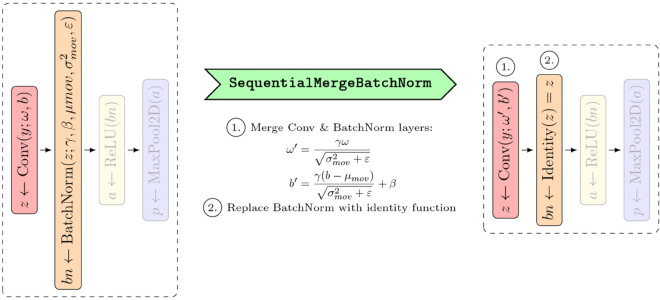
An example of how the SequentialMergeBatchNorm canonizer is used. The canonizer merges batch normalization layers with the linear layers preceding them in a configuration of layers commonly used in models of the VGG family.

Another challenge with rule-based attribution methods, specifically LRP, is the lack of *implementation invariance*. This means that rules of the method may not be directly applicable to specific architectures or yield incompatible results, depending on the model architecture, its implementation, and its learned mathematical function. This issue can generally be solved by converting the model into a canonical form [7,69,70]. For instance, multiple consecutive linear layers with an activation at the end require careful handling for some variants of LRP. This specific scenario requires a merge of the consecutive linear layers into a single linear layer. Recent work demonstrates that appropriate network canonization has perceivably and measurably positive impact on the attribution quality [71,72]. *Zennit* implements several different *canonizers* to temporarily transform models in-place to their canonical form. All of these canonizers derive from the Canonizer class. A common example of a layer configuration requiring canonization in order to achieve optimal attribution quality that is often encountered in NN architectures is batch normalization [73]. Here, *Zennit* provides the MergeBatchNorm canonizer, which temporarily merges the parameters of batch normalization layers into respective adjacent linear layers [74–76], improving the model structure for attribution backpropagation. The MergeBatchNorm canonizer and its extension SequentialMergeBatchNorm can, for example, be used in the VGG family of models [77]. (cf. Fig 7). To offer support for custom and novel network architectures, *Zennit* further provides generalized canonizer implementations. For instance, the AttributeCanonizer temporarily modifies (instance) attributes in-place. This may be used to split a module for which no applicable rule exists, to obtain a workable form in terms of sub-modules. One use-case of the AttributeCanonizer is to optimize the results of an LRP application on ResNets [78,79] by exposing the residual connection in order to attribute it as a weighted sum, similar to how average-pooling is handled. Specifically, the forward method is temporarily overwritten to utilize an explicit Sum module, which can be attributed, instead of the built-in addition, which cannot be attributed, as it is not a module. To further simplify the attribution process for popular architectures, *Zennit* offers model-specific Canonizers for widely-used models such as VGG [77] and ResNet, e.g. as provided by Torchvision [80]. Canonizers can be used on their own, or more conveniently managed by composites. In this case, Canonizers are applied immediately before rules are mapped and registered to the layers during the registration process of the composite to a model.

### Attributors

Attributors provide an additional and optional abstraction layer encompassing all the forward and backward passes of the model required to compute specific feature attributions. The simplest Attributor is the Gradient attributor, which simply computes the gradient by performing a forward- and a backward-pass. Attributors are also used to implement black-box attribution approaches such as SmoothGrad, Integrated Gradients, and Occlusion. Any Attributor can be optionally provided a composite, which will be registered automatically during its execution. The most common approach to compute rule-based feature attributions in *Zennit* is to use any desired Composite with the Gradient Attributor. Providing a Composite to any gradient-based black-box attribution approach, such as SmoothGrad, will modify the gradient utilized by the black-box approach. The result of this will be a combination of these two methods, i.e. a stochastically *smoothed* version of LRP. *Zennit* also implements several black-box attribution methods that are not based on gradient-like computations, such as Occlusion Analysis [34]. However, the combination of such methods with Composites does not change the result, as they do not make use of any gradients to begin with.

### Heatmaps

**Fig 8:**
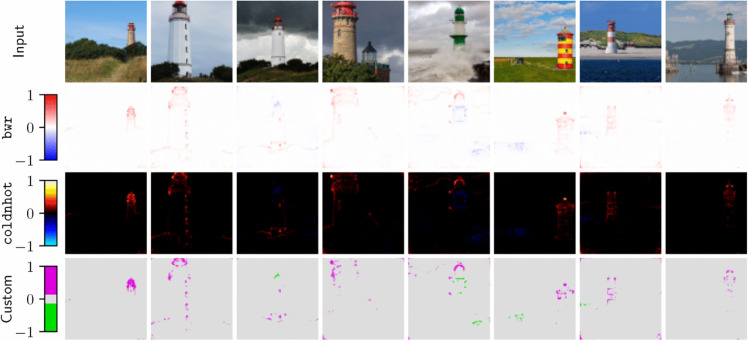
Heatmaps of attributions of lighthouses, using the pre-trained VGG-16 network provided by Torchvision. The composite EpsilonGammaBox was used for computing attributions. Each row after the inputs in the top shows a different color map natively supported in *Zennit*. The meaning of each color value are shown at the left of each row, e.g., for coldnhot, negative relevance is light-/blue, irrelevant pixels are black, and positive relevance is red to yellow). The *Custom* color map show-cases the color-map specification language, which also supports discrete color-maps. The code for the custom color-map is ’70:0d0,70:ddd,90:ddd,90:d0d’.

As attribution scores for image data are often visualized as heatmaps, *Zennit* offers an image module, which can be used to translateattributions scores to color values for visualization as heatmap images, implementing various color maps commonly employed in literature. In order to support the convenient customization of heatmap visualizations *Zennit* provides a *Color-Map Specification Language*, used to specify color maps via short string sequences of (optionally indexed) hexadecimal values. The image module of *Zennit* stores heatmap images using intensity indices coupled to 8-bit color palettes. Here, the intensity indices correspond to attribution scores, enabling a trivial exchange of color palettes in order to obtain different visualizations for heatmaps already saved to disk, avoiding the need to re-compute attributions at later points in time. Examples for heatmaps of attribution scores using different color palettes, computed and visualized with *Zennit*, are shown in Fig 8.

### Practical application example

Listing 1 shows a code example of a typical application of *Zennit* on the Torchvision VGG16 model with batch normalization. The LRP attribution is specified by passing the low and high keyword arguments for configuring the box rule ZBox (i.e. the lowest and highest possible input value, see Eq. 9) as well as the required Canonizers (here SequentialMergeBatchNorm) to the built-in EpsilonGammaBox composite. The now configured composite is then passed together with the model to the Gradient attributor. For more code examples, how-to articles, and an in-depth tutorial on *Zennit*, we refer to the documentation (https://zennit.rtfd.io/en/1.0.0/getting-started.html).

**Listing 1**. Example Python code to compute LRP attribution scores of random data of Torchvision’s VGG16 model with batch normalization. The SequentialBatchNorm canonizer merges the batch normalization into adjacent linear layers, immediately before the EpsilonGammaBox composite is applied. The Gradient attributor computes the gradient, which is modified by the composite within its context, resulting in the computation of LRP attribution scores with the best-practice EpsilonGammaBox ruleset.



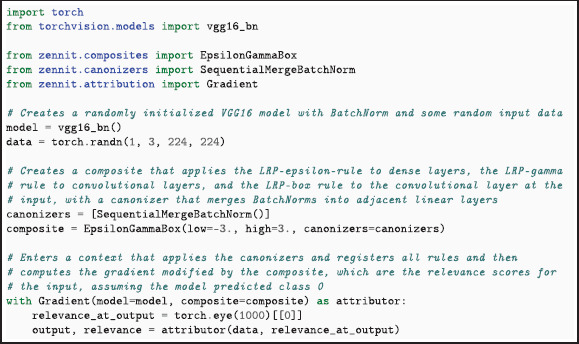



## Building analysis pipelines with CoRelAy

Attribution methods on their own can provide qualitative and quantitative insights into the prediction of a model given individual samples. However, in order to uncover general strategies in model behavior, either comprehensive manual and labor-intensive analysis is necessary, or one can use suitable tools to systematically automate such an explanation-based dataset-wide analysis robust against human error. To this end, [20] introduced Spectral Relevance Analysis, with which they quantitatively analyze a model’s prediction strategy by clustering attributions using Spectral Clustering [81,82] and using embedding approaches such as t-distributed Stochastic Neighborhood Embedding (t-SNE) [83] in order to visualize the results. Anders et al. extended SpRAy with canonical improvements that more closely integrate the visualizations with the analytical results. They also proposed pre-ranking scores for attribution structures to highlight interesting classes discovered during analysis, thereby increasing the reliability of the method and further reducing the human workload [21]. In this section, we introduce *CoRelAy*, which is a tool to quickly compose quantitative analysis pipelines as required by SpRAy, providing multiple embeddings, representations, and clustering labels for the data. While our main use-case and motivation for *CoRelAy* is the analysis of attributions provided by *Zennit*, *CoRelAy* is not limited to any particular kind of data. For instance, *CoRelAy* may also be used to perform a quick dataset exploration with multiple clusterings and embeddings.

### Processors and params

In *CoRelAy*, *processors* are the actions within a pipeline. To create a custom action, a subclass of the Processor class must be implemented. This subclass typically contains class attributes of type Param, which defines hyperparameters of the action, as well as a method with the name function which implements the action. In Python terminology, Params are descriptors, which change based on the instance they are bound to (similar to methods). Params are used to easily define the arguments of Processors, their desired types, and default values among others. The Processor base class contains the Param is_output, which is used to specify whether the output of this processor is an (intermediate) output of the Pipeline. Additionally, the Processor base class contains a Param called io, which can be assigned a Storage object for caching the result of the processor on disk. *CoRelAy* provides a plethora of built-in processors, that are categorized into *pre-processing*, *distance functions*, *affinity functions*, *Laplacians*, *embedding methods*, and *flow processors*. Flow processors can be used to design complex *pipelines*. The most important flow processors are Parallel and Sequential. The use of Parallel allows the output of the previous processor to be passed to multiple other processors. This can be used to compute multiple clusterings, lower dimensional embeddings, etc., using different hyperparameters (i.e. data-parallel, not process-parallel). The use of Sequential allows a sequential combination of processors to split an action into multiple steps within a single Task of a Pipeline.

### Pipelines and tasks

In *CoRelAy*, Pipelines can be seen as computational templates, where each step can be changed individually to customize the result. These steps are implemented as Tasks. Each Task contains a default Processor and an optional *allowed type* of Processor to limit the family of possible actions. During the instantiation of a Pipeline, Tasks are filled with new Processor instances to change their related action from the default one. Pipelines are executed through the function call syntax of Python, where the input data is provided as arguments. Depending on the Processors and states of the is_output flags, the output of the Pipeline consists of zero, one, or a hierarchy of results. If Processors within the Pipeline own an io object, their results are cached and identified through hashing of the input data and parameters. Calling the same Pipeline twice with the same data results in no re-computations during the second execution, as the output data is loaded from the io object. *CoRelAy* implements a SpRAy Pipeline that implements a process akin to the experiments by Anders et al. [21]. This implementation produces data which can be directly used with *ViRelAy* for visualization. An example for the instantiation and execution of a SpRAy pipeline is shown in Listing 2.

**Listing 2**. Example code demonstrating the instantiation and execution of a simple SpRAy pipeline, expecting 8 eigenvalues for the Spectral Embedding. The embeddings are clustered using k-means with k∈{2,...,20} and visualized using t-SNE. Additionally, the results are cached in a file named spray.h5.



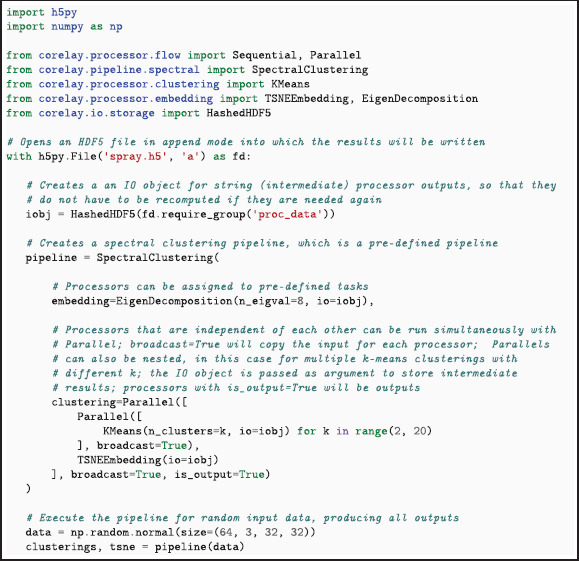



## Interactive visualization with ViRelAy

A quantitative analysis, as conducted through *CoRelAy*, can result in a large amount of various results and representations to the original data, the analysis of which may encompass a significant amount of labor. Here, a manual comparison of individually created plots is inevitable to extract the essence of the results and reveal particularities in the data. Specifically, SpRAy produces a very distinct and common set of objects that need to be compared: the source data points, their attribution scores (with respect to (w.r.t.) a model), a 2-dimensional representation of the (embedded) attribution scores, clustering labels, and global auxiliary scores. In this section we introduce *ViRelAy*, an interactive web-application which visually connects the aforementioned five objects to allow a free and intuitive exploration of the analysis results. The backend and frontend of *ViRelAy* are implemented in Python using Flask [84] and Angular [85] using TypeScript [86], respectively.

### Data loading.

*ViRelAy* is designed to process the data of *CoRelAy*. The results of *CoRelAy* are stored as HDF5 [87] databases in a hierarchy which can be used by *ViRelAy* statically, reducing loading times for an improved user interaction quality. A *project file* references this HDF5 analysis database, as well as the source data and the attribution data, which are stored in separate HDF5 databases. A single project file contains exactly one source dataset with one respective dataset of attribution scores, as well as an arbitrary amount of analysis files. To compare different datasets or attribution approaches, *ViRelAy* can be provided with an arbitrary amount of project files, between which the user can switch during their interaction at runtime.

### Explorative user interaction.

**Fig 9:**
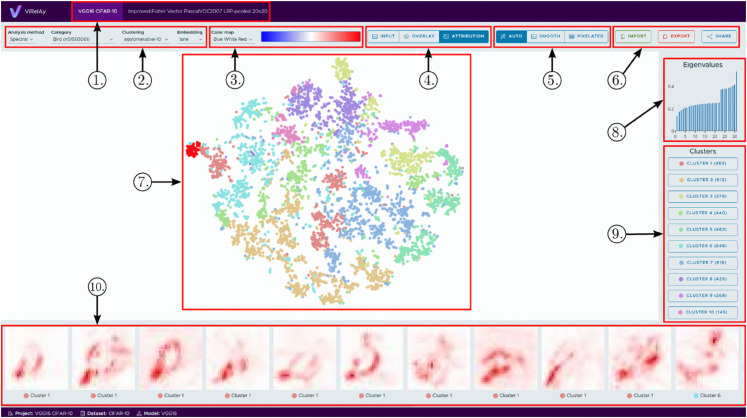
The *ViRelAy* user interface. Highlighted points are: (1) Project selection, (2) analysis setup and category selection, (3) color map selection, (4) data/attribution visualization mode selection, (5) image sampling mode selection, (6) import/export/share current selection, (7) 2D visual embedding canvas, (8) auxiliary score plot, (9) cluster point selection, and (10) data/attribution visualization.

The user interface of *ViRelAy* is shown in Fig 9. At the top of the interface is (1) the project selection, where the projects, as defined in the project files, show up as tabs and may be selected to switch between datasets and attribution methods. Below the project selection, on the left side is (2) the analysis selection, where the analysis approach (given by supplying multiple analysis files in a single project file), the category (which is often a class label, but may be any kind of grouping of data points chosen by the project creator), the clustering method (which influences (9) the available clusters and (7) the data point coloring), and the embedding (which is the 2D representation of the data points as shown in (7) the visualization canvas) can be selected. Selecting a different analysis method resets all other settings. To the right is (3) the color map selection, which changes the color map used in (10) the data/attribution selection, with a color bar indicating low (left) and high (right) values. The next item to the right is (4) the data/attribution visualization mode selection, which changes whether (10) the data/attribution visualization shows the source data (input), its attribution scores with the selected color map (attribution), or the attribution scores superimposed onto a gray-scale image of the source data (overlay). To its right is (5) the image sampling mode selection, which determines how the browser displays images in (10) the data/attribution visualization. *Smooth* will use a smooth sampling method like cubic interpolation, while *pixelated* will use a sharp sampling method, like nearest neighbor interpolation. The *smooth* mode is used for larger images, while the *pixelated* mode is used for smaller images. This makes it easier for users to inspect samples and heatmaps without losing important details: when the sample images and heatmaps are small, smoothing the image could potentially smear out important details, while pixelating large images could potentially hide small details, because lines of pixels are skipped. The *auto* mode will switch between *smooth* and *pixelated* based on the size of the images. The (6) *import* and *export* buttons allow to export the currently selected analysis, category, clustering, embedding, color map, visualization mode and selected points by downloading a JSON-file [88,89], or importing a JSON-file to change the selections to the configuration of a previously exported file. This may be used either to store or to share interesting results. The selection may also be shared or bookmarked in the form of a URL using the (6) *share* button. At the center of the interface is (8) the 2D-visualization canvas, which shows the points in the selected 2-dimensional embedding space (produced by, e.g. t-SNE) colored by the clusters indicated in (9) the cluster point selection. In this canvas, the user may zoom or pan, and select points which will be highlighted by a more saturated color and shown in (10) the data/attribution visualization. Hovering over data points will show a preview of the source data inside the canvas. To the right is (8) the auxiliary category score plot, which in this demonstration are the eigenvalues of the Spectral Embedding. Below, there is (9) the cluster point selection, which shows the available clusters of the selected clustering, as well as the colors used for members of these clusters in (7) the 2D-visualization canvas, and the number of points in this cluster in parentheses. Finally, at the bottom is (10) the data/attribution visualization, where, depending on which mode was selected in (4) the data/attribution mode selection, will show either the source data, the attribution score heatmap, or the attribution scores superimposed on a gray-scale version of the source image of a subset of the selected points.

## Comparison to alternative frameworks

To put our work into context, we provide a comparison of each of our software packages to available alternatives. We compare *Zennit* to similar attribution frameworks, with a focus on propagation-based approaches, in particular LRP. Although *CoRelAy* is a domain-specific framework, we compare it to a few alternatives with increasing complexity. Since *ViRelAy* solves a very specific visualization problem for which no real alternatives exist, we provide an overview of other XAI visualization applications.

### Attribution frameworks.

With the growth of the field of XAI, numerous explainability and feature attribution frameworks beside *Zennit* have emerged for various areas of application. Some frameworks (e.g. Captum Insights) even provide visual frontends to enhance interpretability. Although various frameworks seem to solve similar challenges, some of them did not stand the test of time, and ultimately became unmaintained shortly after their publication. Table 1 lists some popular attribution frameworks, along *Zennit*, with columns that focus on the main objective of *Zennit*: to provide a feature-complete, modular and customizable framework for propagation-based attribution methods with a focus on LRP and with additional general attribution method capabilities. Many frameworks were primarily designed for the examination and explanation of classical or white-box (glass-box) methods (e.g. interpretML [66], explainerdashboard [67], alibi [90]). While some frameworks exhibit some overlap with approaches commonly used for DNNs, we do not list these here due to their limited comparability.

The framework that is most comparable to *Zennit* is *iNNvestigate* [57], which provides a feature-complete implementation of LRP, including various common and recommended backpropagation rule composites for TensorFlow [58] and Keras [59]. While it is well suited for LRP on models implemented in Keras, a lack of an easily configurable interface to implement custom rules or rule-maps makes it less efficient to apply and adapt to novel models and architectures. Although iNNvestigate is next to *Zennit* the only other framework with any rule-mapping capabilities , only *Zennit* provides *canonization* to adapt models architectures on the fly to optimize the applicability of rule-based attribution methods. Examples and tests with CI, as well as a basic usage in the readme and an API reference is provided with iNNvestigate.

*Captum* [16] implements several commonly used attribution approaches in PyTorch, which makes it superficially comparable to *Zennit*. While it provides a wide collection of methods, *Captum* only supports simple propagation-based attribution variants, relating to its limited implementation of LRP: currently only ${\text{LRP}}_{\varepsilon }$ is supported, and unfortunately, no interface for custom rules, or mapping rules to parts of the model architecture, exists. Since there is no support for LRP rules other than ${\text{LRP}}_{\varepsilon }$, support for model canonization also is not implemented.

*TorchRay* [36] is another alternative that implements attribution methods in PyTorch. It does not support any propagation-based approaches except for Guided Backprop and similar methods based on (unmodified) gradient computation. Although other attribution methods are supported, most notably RISE [35], the project is currently unmaintained since October 2019.

Finally, *DeepExplain* [60] provides another alternative for Keras-based XAI, yet only supports ${\text{LRP}}_{\varepsilon }$ and DeepLIFT for propagation-based attribution. While other attribution-based approaches are available which are not supplied by iNNvestigate and tests with CI are implemented, its documentation is limited and the framework is currently unmaintained since August 2020.

A common theme among the discussed attribution frameworks is their inflexible architecture, allowing them to only implement simple attribution methods with relative ease, while more complex rule-based methods are either hard or even impossible to implement. In contrast, *Zennit* has already proven its flexibility and versatility by serving as the foundation for the design of novel XAI approaches, one notable example being the implementation of Concept Relevance Propagation (CRP) [27], depending upon and extending the feature set of *Zennit*. Beyond this, *Zennit* has been extensively used in various highly involved experimental setups, including feature attribution for regression problems [24], debugging and improving NNs [21,25], preventing catastrophic forgetting through relevance-based neural freezing [26], concept-based attribution [28], improving MPRTs [29], model quantization [30], applications in histopathology [31], clinical gait analysis [32], a novel relevance-based alternative to gradient descent [33], and in many other notable works [93–97].

### Pipelining frameworks.

The primary objective of *CoRelAy* is to enable the development of analysis pipelines for attribution data from local XAI methods, aiming to generate data which seamlessly integrates with *ViRelAy*. Although *CoRelAy* was particularly designed with this specific use-case in mind, we compare it to other frameworks w.r.t. a re-implementation of the same workflow using alternative pipelining frameworks. Since *CoRelAy* uses implementations provided by *Scikit-Learn* [63] for some pipeline steps (e.g. t-SNE and k-means), a logical alternative would be to directly implement SpRAy using Scikit-Learn’s native pipelining framework. Similar to *CoRelAy*, Scikit-Learn’s pipelining framework is optimized for single machine pipelines implemented in Python. Both frameworks provide functionalities to cache intermediate results. The most obvious downside of using Scikit-Learn’s pipelines is the increased implementation cost for SpRAy and the necessary implementation of the interface to *ViRelAy* .

*Luigi* [64] offers a more advanced pipelining framework specifically made for long-running batch jobs. While *CoRelAy* and Scikit-Learn generally use parts of computations as tasks in a single pipeline, Luigi is positioned one layer of abstraction higher. Here, it delegates (usually thousands of) tasks to multiple pipelines, which are not necessarily only computations in Python. Luigi provides a client-server model, where a central server schedules tasks executed by clients. In addition, a web server is built into Luigi to visualize the dependency graph of the pipeline. While smaller pipelines like SpRAy can be constructed and executed using Luigi, the computational complexity of these pipelines, even on datasets as large as ImageNet [23], is usually low enough that they can be executed on a single machine, which defeats the only major advantage of Luigi over using *CoRelAy* or Scikit-Learn.

For even more advanced, distributed pipelines, *Apache AirFlow* [65] can be utilized to develop, schedule, and monitor complex batch-jobs. Although similar to Luigi in functionality, AirFlow provides a large amount of interoperability and integration for distributed and high-performance computing, as well as high scalability. Although it is suitable for both large and small workflows, including SpRAy, the added code complexity may outweigh its benefits, especially for small, single-machine workflows.

### XAI visualization applications.

*ViRelAy* addresses a specific issue by visualizing a set of related analysis results of different data domains. In particular, these results are constituted of image samples along with auxiliary visual representations (here attributions), a 2D-representation which allows an easy comparison of samples, as well as multiple color-coded clusterings or labelings. While various applications can be addressed using *ViRelAy*, its primary focus is the visualization of the embeddings and clusterings of attribution representations obtained from the SpRAy method. In light of its application-specific nature, there are no true alternatives. However, there are other software packages available that can assist users in the examination of models using feature attribution or other approaches of XAI.

*Captum Insights* [16] is a web interface incorporated into Captum which enables the visualization and interaction with data samples, model predictions, and feature attributions. Although it lacks the capability to visualize embeddings or clusterings, it enables the presentation of data samples alongside their attribution scores and prediction probabilities for various classes. This superficially resembles the feature set of *ViRelAy*, albeit less comprehensive. The provided visualization interface of Captum Insights is mainly static, leading to a somewhat limited level of interaction.

The *interpretML* [66] framework is mainly designed around fitting *glass-box* (i.e. inherently interpretable, non-black-box) models, while also providing a few post-hoc explanation methods. Users can use its dashboard feature to visually explore individual samples, respective feature importance scores and the detailed performance of the model. While both interpretML’s dashboard and *ViRelAy* offer the visualization of feature importance, *ViRelAy* places a stronger emphasis on the *analysis* of the feature importance rather than visualizing model performance and predictions.

*Explainerdashboard* [67] provides a similar set of explainability methods wrapped into a single, Scikit-Learn-compatible interface. This interface directly executes a dashboard, providing a detailed overview of an analysis of the model. In addition to feature importance, feature dependence, and feature interactions (provided through Shapley Values [91,92]), the dashboard visualizes statistics over the model performance and predictions for specific samples. Furthermore, a sample perturbation interface to analyze the prediction under specific changes to individual samples is included. Specifically for random forests and XGBoost models, the dashboard features a view of the individual decision trees. Based on these distinctive features, Explainerdashboard offers an effective interaction to investigate simple models trained on tabular data. However, there is no support for the visualized feature analysis of image data as implemented in *ViRelAy*.

## Dataset-wide Explainable AI

In this section, we demonstrate how results produced by *Zennit* and *CoRelAy* can be analyzed using *ViRelAy* to discover CH behavior in a model’s predictions. For a technical description of the creation of a *ViRelAy* project, we refer to Appendix Creating a *ViRelAy* project.

### Analyzing classifiers and datasets.

Lapuschkin et al. [20] performed SpRAy on a Fisher vector classifier trained on the PASCAL VOC 2007 dataset and found CH-inducing spurious correlations in the dataset. We will recreate their analysis and demonstrate how the use of *ViRelAy* streamlines the process of identifying such defects in an intuitive manner. A complete guide for this analysis can be found in our documentation (https://virelay.rtfd.io/en/1.0.0/user-guide/how-to-analyze-classifiers-and-datasets.html).

**Fig 10:**
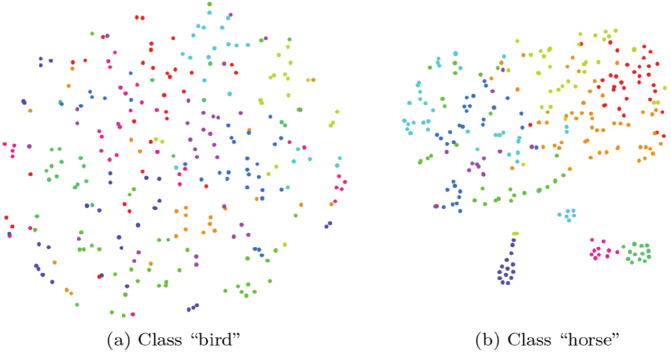
Comparison of t-SNE embeddings of classes “bird” (left) and “horse” (right). Each data point represents a spectral embedding of an attribution of a sample from the dataset that was projected into 2-dimensional space using t-SNE. The colors indicate the different clusters identified by the currently selected clustering method.

We use a SpRAy pipeline that, based on the samples in input space, produces t-SNE (as seen in Fig 10) and spectral embeddings of the attributions, as well as clusterings (e.g. *k*-Means), similar to the implementation in Listing 2. The samples are categorized by their Pascal VOC 2007 class. At the beginning of an inspection with *ViRelAy*, it can be beneficial to obtain an overview of the various embeddings and clusterings. Depending on the problem setting, different choices of embedding and clustering type may offer more valuable insights compared to others. In this project, we found the t-SNE embedding, which is in turn based on the spectral embedding of the attributions, to be most informative. Consequently, we performed a more in-depth examination of this representation in order to identify outlier clusters. Especially small outlier clusters may indicate potential CH behavior, suggesting that the associated prediction strategy was learned for only a small subset of training samples. These samples may share a specific feature that was exploited by the classifier. Indeed, when exploring the t-SNE embeddings, it can be noted that some classes exhibit highly homogeneous embeddings, whereas others contain one or more outlier clusters. For instance, Fig 10 shows a comparison of the t-SNE embeddings for the classes bird and horse.

**Fig 11:**
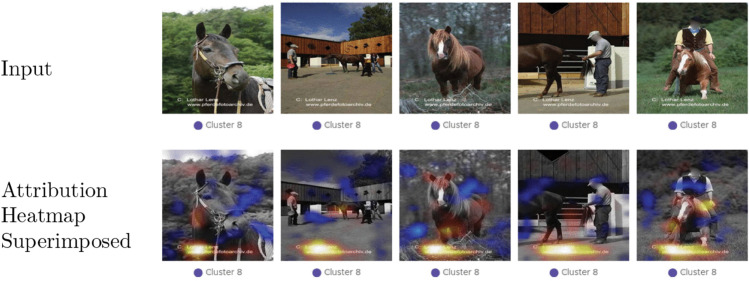
Input images and their respective feature attributions. Top: input images of the samples in an outlier cluster of the class horse; Bottom: gray-scale versions of the same images with the attribution heatmap superimposed onto them (sample viewer with display mode *overlay*). Positive relevance increases from red to yellow to white color. Negative relevance increases from blue to cyan. Reprinted from http://www.pferdefotoarchiv.de as part of the PASCAL VOC 2007 dataset under a CC BY license, with permission from Lothar Lenz, original copyright Lothar Lenz 2007.

We can observe that the t-SNE embedding for the class *bird* is highly homogeneous, indicating that the attributions are broadly similar. In contrast, the t-SNE embedding for the class *horse* exhibits multiple outlier clusters, which can be seen at the bottom of Fig 10b. This signifies that the attributions for the samples contained in these outlier clusters may be dissimilar to the attributions of the larger main cluster, suggesting that the classifier has learned multiple distinct classification strategies for a specific subset of samples in the horse class. This warrants a further manual investigation of the samples in question. A visual inspection of a few training samples from the drop shaped outlier cluster at the bottom are shown in Fig 11 (top). Here, we notice a copyright notice watermark at the bottom of the images, which is a feature that all samples of the outlier cluster have in common.

To verify whether this specific feature caused these samples to be clustered together, we can examine the associated attribution scores. *ViRelAy* enables users to directly view the attribution scores in the form of plain heatmaps, or as heatmaps superimposed onto the input images. Consequently, we can straightforwardly correlate the attribution scores with the underlying image features. Attribution scores become difficult to view in overlay mode when they are too delicate, in which case they are better directly examined. Conversely, when the attribution scores are coarse, it may be harder to connect the attribution scores in the heatmap to the features in the corresponding image regions. Therefore, the overlay mode is best to identify the visual features related to significant attribution scores. In this specific project, we observe the attribution scores to be primarily coarse, consequently the overlay mode is used for visualization. These visualizations as well as their corresponding original image samples are depicted in Fig 11 (bottom).

The attribution scores suggest that the classifier primarily bases its classification decision on the copyright notice at the bottom of the images. An examination of the other outlier clusters unveils that all of them manifest the same artifact, where each cluster contains a slightly different copyright notice. This is an indication that the classifier exhibits CH behavior for the class horse. Although this particular finding was already known, we were able to demonstrate the practicality to identify such CH behavior in predictors using *Zennit*, *CoRelAy*, and *ViRelAy*.

### Verifying SpRAy on CIFAR-10.

In order to verifying whether the implementation provides reasonable results, we conduct a benchmark experiment on CIFAR-10.

*Setup:* For each of the 10 classes in the dataset, we create one 2-class classification setting with the *previous* class. E.g., the setting for class 1 is a 2-class classification between class 1 and class 0 (for class 0, we wrap around to class 9). For each of these 10 resulting settings, we train two models: one “clean” control model, in which we simply train a convolutional neural network on the unmodified data, and a poisoned model, in which we poison 50% of the samples of the latter class with a constructed artifact in the form of a 2 by 2 gray pixel box in the top left of the image. We evaluate the accuracy on a modified test set, where we instead poison all samples of the class which was not poisoned during training. Furthermore, we follow [21] and produce clusters using SpRAy, where we compute attributions using the Epsilon-Gamma-Box in *Zennit*, and use spectral HDBSCAN implemented in *CoRelAy*. We conduct 5 trials of each experiment, where each trial corresponds to a specific model initialization.

*Expected outcome:* Given that the poisoned model exploits the artifact, we expect to see the test accuracy to be consistently worse for the models trained on the poisoned data compared to the control models. This indicates CH behavior of the poisoned model, which is what we try to identify using SpRAy. Anders et al. [21] use the linear separability of SpRAy clusters (called “tau score”) as a weak indicator of CH behavior. Therefore, we expect to see a higher tau score for models exhibiting CH behavior. In summary: if we see the accuracy on the (inversely poisoned) test set to be consistently lower (i.e, the model exploiting the artifact and thus showing CH behavior), we also expect the linear separability of the SpRAy clusters (tau score) to be higher.

**Fig 12:**
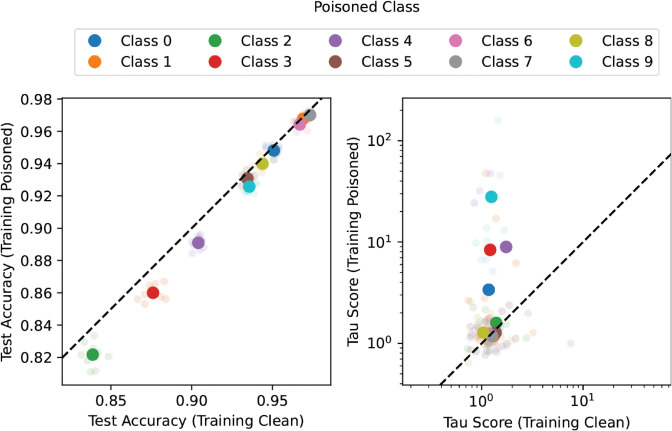
Test accuracies and tau scores on CIFAR-10. Left: Test Accuracy on poisoned training set (vertical axis) vs. clean training set (horizontal axis). Right: Tau-score [21] of HDBSCAN-SpRAy on models trained on poisoned data (vertical axis) vs. models trained on clean data (horizontal axis). The light, small dots are individual trials (models) and the larger, thick dots are the empirical mean over the trials for that particular color. Each color represents the class which was poisoned each setting, where each setting is a 2-class classification with the previous class (e.g., class 0 means the classification was class 9 versus class 0, where class 0 was poisoned). The dashed line visualizes the points on which poisoned and clean training accuracy would be equal.

*Results:* Fig 12 left shows a scatter plot of the (inversely poisoned) test accuracies on the control model (horizontal axis) compared to the poisoned model (vertical axis). As the dashed black line indicates equal accuracies between these models, and we can see all models consistently below this line, we can confirm our expected outcome that the models trained on the poisoned data exhibit CH behavior. Fig 12 right shows a scatter plot of the linear separability of the clusters found through HDBSCAN-SpRAy (tau-score) in the poisoned class on the control model (horizontal axis) compared to the poisoned model (vertical axis). Values above the dashed line indicate a larger tau score for poisoned models. While not as consistent as for the accuracies, we see that some setups show a considerable increase in the tau score of the poisoned model compared to the control one. This is in line with our expected outcome.

## Conclusion

In advocacy of reproducibility in ML [98], we have introduced three open source software packages to attribute, analyze, and interactively explore a model’s dataset-wide prediction strategies. With *Zennit*, we hope to provide an intuitive tool within the boundaries of PyTorch to compute attributions in a customizable and intuitive fashion, and to make the multitude of rules in LRP and other rule-based attribution methods more accessible. We especially hope that this enables the analysis of any kind of model through the streamlined process of extending attribution approaches based on the intuitive structure of *Zennit*. Through *CoRelAy*, we hope to provide a simple way to analyze attributions dataset-wide in swiftly built pipelines, and thus explore the unused potential of insight into prediction models. Using *ViRelAy*, we hope to make the exploration of analysis results as effortless as possible by providing an interactive combined viewer of source data, attributions, visual embeddings, clusterings, and others. *Zennit*, *CoRelAy*, and *ViRelAy* in combination have already been successfully used in the analysis of ImageNet on millions of images to find artifactual CH behavior [21], thus demonstrating effectiveness and scalability. With the introduction of these software packages, we hope to aid the community in the research and application of methods of XAI and beyond, to gain deeper insights into the prediction strategies of DNNs.

## Appendix

### Testing and quality assurance

To ensure a high quality of our XAI tools, we have created a comprehensive testing framework, which covers all aspects of the source code in order to make the software packages more robust, reliable, and maintainable. This includes a suite of unit tests, static code analysis tools, and a CI pipeline.

Each of the software packages has a comprehensive unit test suite written using the PyTest framework [99], which is designed to test every line of code to verify its correct functioning and accuracy. We are always aiming at achieving 100% test coverage, not only in terms of line coverage, but also in terms of branch coverage. This ensures that each line and branch of the source code is executed in at least one test. Although full test coverage does not necessarily translate to having covered all possible cases and eventualities, it is still a desirable goal and a useful approach to code quality, as it guarantees that each software component is thoroughly vetted and validated. Furthermore, our comprehensive test regime allows us to make changes to the code base and release new versions with confidence, as it ensures that changes or additions do not break any existing functionality.

We are not only committed to producing high-quality software, but also to build a healthy community around them. Among other things, this means that we not only accept but encourage community contributions. Working in a large, diverse, and distributed team means that we have to take utmost care of code quality beyond just testing its correct functioning. This includes but is not limited to: (1) common code style guidelines, (2) the usage of well-established programming patterns, and (3) the avoidance of anti-patterns and common pitfalls. To this end, changes are introduced via pull requests and undergo a manual code review, before they are being merged into the main code base. In aid of this effort, we employ static code analysis tools, to automate part of the code review to the greatest extent possible. These automated inspections scrutinize the code for adherence to established best practices, such as naming conventions, coding style and logical program structure, and check the code for common code defects, security issues, and other code smells that may hint at deeper problems.

Specifically, we utilize PyLint [100], as well as PyFlakes [101], PyCodeStyle [102] and McCabe [103] through Flake8 [104]. PyLint is a linter for the Python programming language that checks the code for errors, and adherence to coding standards and best practices. It can also suggest refactorings to improve code quality. PyFlakes can check Python scripts for errors and is similar to PyLint, but it does not check code style and is more limited in its understanding of types, as it only analyzes the script’s syntax tree. This, however, makes it much more performant in comparison to PyLint. PyCodeStyle is a code style checker, which checks Python code against a subset of the style conventions laid out in PEP8 [105]. McCabe is a tool that is solely used to compute the McCabe complexity [106] of the source code, which is also known as cyclomatic complexity and measures how complex a program is. A high McCabe complexity hints at poor software design, which in turn reduces the readability of the code and makes it harder to make changes to it. Generally, a high McCabe complexity indicates that the code or parts thereof should be refactored. Flake8 combines PyFlakes, PyCodeStyle, and McCabe in a single tool and thus makes it easier to use them.

Static code analysis does not only help with the code review process, but can also be incorporated into the development process to continuously inspect the source code and rectify issues as they arise. By incorporating static code analysis tools into our development process, we not only improve code readability and clearness, and make it easier to work in a team, as everyone adheres to a standardized coding style, but we also reduce the risk of introducing defects or ambiguities into our code base.

To further ensure the integrity of our software, we have implemented a CI pipeline that automatically runs a series of tests whenever code is pushed to the master branch or a when a pull request is merged into the master branch of our GitHub repository. This CI process verifies each change to our code base, to ensure that that it does not break existing functionality and adheres to our established coding standards. The unit test suite is run on a matrix of different Python versions to verify that the software packages work as intended on a variety of Python versions commonly used in software projects. This is achieved using tox [107], which is a tool that standardizes testing in Python and serves as a frontend for CI servers. Its goal is to reduce the effort required to configure a test environment both on a local machine, as well as on a CI server. Among other things, it enables users to install a package and run tests on it in different Python environments. By integrating testing and validation directly into the development workflow, we minimize the likelihood of errors propagating through the system and ensure that our software remains robust and reliable throughout its lifespan.

Through the use of unit tests and static code analysis tools, and the constant validation through the CI pipeline, we have created a rigorous quality assurance framework that promotes the quality, reliability, and maintainability of our XAI tools. By leveraging these tools and techniques, we not only ensure that our software meets our standards of quality, but also make certain that a healthy community of contributors can grow around the project without comprising the quality of our code.

### Feature attribution approaches

This section provides a high-level description of some common feature attribution approaches.

#### Perturbation analysis methods.

*Occlusion Analysis* [34], is a method for determining a high-level understanding of the importance of regions of an input image by measuring the impact of occluding these regions on the classification result.

*RISE* [35] is another perturbation-based method that, instead of occluding specific image regions, generates numerous random masks to perturb the input image in question. Then a saliency map is generated, which is a linear combination of the random masks with the model’s output at the target class as weights. Not only does this method produce more detailed saliency maps than other methods, but authors also show that the number of salient pixels that have to be removed in order for the model to change its classification decision is lower than for any of the methods they compare against.

Many perturbation-based methods try to find a mask for the input image that both maximizes the output of the model and minimizes the number of pixels preserved by the mask, while penalizing irregular mask shapes. The proportion between these three optimization goals are identified by Fong et al. as a problem with existing methods, because choosing different parameters for the trade-off produces different masks, where superiority of any of the resulting masks cannot be established. To rectify this situation, they propose *Extremal Perturbation* [36], which instead constraints the area of the mask to a fixed fraction and uses the model’s output as an optimization target.

#### Sensitivity analysis methods.

*Guided Backprop* [37] is a technique where the gradient of the model is selectively backpropagated, i.e. guided through the model. The gradient of ReLU [108,109] activation functions is only passed through if both the gradient and the activation of the neuron are positive and non-zero. This results in a sensitivity map, which highlights input features that, if increased, would have the most positive influence on the prediction outcome, thus providing a measure of feature importance.

Sundararajan et al. propose two fundamental axioms for attribution methods: (1) *Sensitivity*, which is satisfied when the method assigns a non-zero attribution to differing features in inputs that have different predictions, and (2) *Implementation Invariance*, which is satisfied when the method assigns identical attributions for functionally equivalent models that differ in implementation. They show that most existing methods violate these axioms. To address this, they developed *Integrated Gradients* [38], a novel method that calculates the path integral of gradients between a baseline input and the target input. Baseline inputs are domain-specific *“null”* elements, serving as reference points for comparison.

Dhamdhere et al. build upon Integrated Gradients by introducing *Conductance* [39,40], a measure that quantifies the attribution flowing through each hidden unit. They argue that conductance is positively correlated with importance for the prediction and provide both theoretical support and empirical evidence. Specifically, they demonstrate that removing hidden units with high conductance has a significant impact on the prediction of the model.

In Convolutional Neural Networks (CNNs), the final convolutional layer produces feature maps that encode information about all features detected in the input image. *Grad-CAM* [41] uses the gradient of the output neuron of interest to explain the CNN’s decision by propagating it back to the last convolutional layer. Multiplying its feature maps by the gradient extracts features relevant to the target class. Averaging the feature maps produces coarse sensitivity maps highlighting important parts of the image.

Smilkov et al. developed *SmoothGrad* [42], a method that enhances the interpretability of sensitivity maps by removing noise to visually sharpen gradient-based sensitivity. This technique can be used in combination with other sensitivity map algorithms to produce more informative results.

#### Decomposition-based methods.

*LRP* [9,10] identifies which input features contribute most to a NN’s predictions, both positively and negatively, by propagating the relevance from the output to the input on a per-layer basis using purpose-made propagation rules. Each neuron receives a relevance score, allowing the user to determine its contribution to the prediction. Negative relevance indicates evidence against the prediction. Over the years, numerous propagation rules have been developed, making LRP a powerful, yet complex to use XAI framework.

*DTD* [43] uses first-order Taylor expansions to express each neuron’s output in terms of its partial derivatives w.r.t. the neuron’s inputs, allowing for easy decomposition of their contributions. DTD aggregates the decomposed relevances and propagates them backward, thus redistributing the model’s output to its input variables. Whereas LRP decomposition rules were motivated by an inversion of the directed acyclic graph flow of neural networks computations, DTD provides a theoretical motivation for their formulation. However, it can be shown that specific choices of reference points reduce the DTD decomposition rules to the LRP propagation rules.

*SHAP* [48] is an approach based on Shapley values [91,92], which come from cooperative game theory. Originally devised to determine the fair distribution of a game’s “winnings” amongst its players, Shapley values are used here to attribute predictions to input features. Lundberg et al. also propose two approximation methods: *GradientShap* and *KernelShap*.

*DeepLIFT* [44] backpropagates the contributions of all neurons to every input feature, where the contribution score is the difference between a neuron’s activation and a reference activation. DeepLIFT optionally treats positive and negative contributions separately, enabling the explanation of features that are evidence against the prediction.

*Excitation Backprop* [45] is a method for creating attention maps that highlight the parts of the input a CNN was “focusing” on when making its classification decision. This approach uses a backpropagation scheme where a probabilistic Winner-Take-All process distributes the signal of an output neuron to lower layers, effectively identifying the most relevant neurons given the input signal. Coincidentally, this approach is equivalent to the LRP *Z*^ + ^-rule. Attention maps can be generated at any intermediate convolutional layer and up-sampled using bicubic interpolation. By selecting higher-level layers, performing the expensive backpropagation all the way down to the bottom layer can be avoided, as the impact of layer selection is minimal.

Decomposition-based methods rely on backpropagating relevance from the output neuron of interest to the input layer to explain model decisions. However, Kindermans et al. argue that this approach neglects noise in the data, leading to inaccurate explanations. They illustrate this issue with a toy problem involving a linear regression model and show that previous methods fail to correctly explain them. Gradient-based methods, for instance, use the gradient to explain how the model’s decision changes along the direction of steepest ascent. However, since this direction is often distorted by noise, these methods do not reveal the signal in the data but rather how to extract it. Kindermans et al. propose two new methods: *PatternNet* and *PatternAttribution* to address these shortcomings [46]. PatternNet applies a layer-wise back-projection of the estimated signal to input space, while PatternAttribution exposes neuron-wise contributions of the signal to the classification score. By ignoring noise, PatternAttribution produces much clearer attribution maps.

#### Surrogate model-based methods.

In the context of fully opaque models, for which neither training data nor model weights are available, but that allow for repeated probing, gradient and decomposition-based interpretability methods are inapplicable. In addition to sensitivity analysis, another class of methods has been established for this scenario that uses surrogate models to locally explain a model.

*LIME* [47] is such a method that uses local surrogate models to make black-box models explainable. First, it creates a dataset with perturbed inputs and corresponding predictions from the black-box model. Then the samples are weighted by their proximity to the sample being explained and a small model is trained on the data to approximate the black-box model’s predictions. This model can then be used in lieu of the black box model for explanations. Its accuracy, however, degrades when explaining samples that are significantly different from the original sample of interest.

*Deconvolution* [34] is a method that attaches a deconvolutional NN [110] (an inverse CNN) to each convolutional layer of the model, which map the learned features back into pixel-space. This reveals the feature hierarchy learned by the model: Early layers identify simple features like edges and textures, while middle layers recognize patterns such as fur or mesh. Later layers combine these features to form increasingly complex representations, including entire objects or facial features.

### Creating a *ViRelAy* project

A *ViRelAy* project consists of (1) a dataset, containing the training samples, (2) a label map, mapping between label indices, label names, and WordNet IDs (if available) to display label names, (3) an attribution database, containing the attribution maps computed using *Zennit*, (4) an analysis database, containing *CoRelAy* meta-analysis results, and (5) a project file, containing meta-data and linking the individual files.

#### Input data.

For purposes of this discussion, we assume that a trained model and an accompanying training dataset are already in place. *ViRelAy* supports two different dataset formats: (1) an image directory with sub-directories for each label containing the respective samples, or (2) an HDF5 database, where the input images are either stored as an HDF5 dataset or group. HDF5 datasets are multi-dimensional arrays suitable for input images with the same resolution, which are stored as a single array of shape *samples* × *channels* × *height* × *width* under the key data. HDF5 groups are similar to files in a file system and can therefore be used in cases where the input images have varying resolutions. In this case the samples are also stored under the key data, but as separate datasets of shape *channels* × *height* × *width* inside a group with unique image IDs as keys. The labels are also stored in a dataset or group called label, depending on the storage format of the input images. If the samples are stored in a dataset, the labels are also stored as a dataset. In the case of a single-label dataset, the labels are stored in a dataset of shape *samples*, where each entry contains the label index, or of shape *samples* × numberoflabels using a multi-hot encoding, in the case of a multi-label dataset. Conversely, when the samples are stored in a group, the labels are also stored in a group, where the keys are the IDs of the corresponding input samples and the values are either the label index or a dataset of shape numberoflabels using a multi-hot encoding. The label map is a JSON file containing an array of labels, where each label is represented by an object that contains the label index, the optional WordNet ID, and the label name. A complete specification and examples of a label map file can be found in our documentation (https://virelay.rtfd.io/en/1.0.0/contributors-guide/project-file-format.html).

#### Attribution data (from Zennit).

*Zennit* can be utilized to compute attributions for all samples in the dataset (cf. Appendix Layer-wise relevance propagation: Details). In order for *ViRelAy* to load these attributions, they also have to be stored in an HDF5 database. The format is analogous to the format of the HDF5 database containing the input dataset, where the key of the dataset/group containing the attributions is instead attribution. In addition, the attribution database contains two more HDF5 datasets/groups: (1) label, containing the ground-truth labels of the respective original samples, and (2) prediction, containing the model’s predictions of the original samples. The labels are stored in the exact same fashion as they are stored in the HDF5 database containing the input images. The predictions are always stored as a vector similar to the multi-label case, containing the classification scores output by the model. Each project can only contain attributions for a single attribution method, but it can contain multiple attribution databases (e.g. an attribution database could be created per class).

#### Analysis data (from CoRelAy).

*CoRelAy* can be used to build analysis pipelines, such as SpRAy (cf. Section Building analysis pipelines with CoRelAy), for analyzing attributions as produced by *Zennit* or other attribution frameworks. In order for *ViRelAy* to consume the resulting analysis data, it must be stored in an HDF5 database, as well. The database may contain results from multiple *CoRelAy* analysis pipelines, each of which stored as a group in the HDF5 database, where the name of the group is a unique identifier of the corresponding analysis. Each analysis group may contain multiple sub-keys describing different categories of attributions for which the analysis was performed. Categories may constitute anything that splits up the data in a helpful manner. Usually, one category is created for each class in the dataset, but the data can be also categorized otherwise, e.g. by WordNet IDs or concepts. The category groups contain a dataset index, which contains the indices of the samples that are in the category, and two groups, embedding and cluster, which contain the embeddings and clusterings computed in the analysis pipeline respectively. Each key in the embedding sub-group represents a different embedding method, e.g. spectral embedding or t-SNE. Each embedding can optionally have multiple attributes: (1) eigenvalue, which contains the eigenvalues of the eigendecomposition of the embedding, (2) embedding, which is the name of the base embedding, if the embedding is based on another embedding, and (3) index, which are the indices of the dimensions of the base embedding that were used. Finally, the cluster sub-group contains the clusterings that were used to cluster the attributions. Each key in the cluster sub-group represents a different clustering method with different parameters, e.g. different values of *k* for a *k*-means clustering. Each clustering can have additional attributes, e.g. embedding, which is the embedding that the clustering is based on, or the parameters of the clustering algorithm.

#### Project file (for ViRelAy).

Finally, these database files are combined in a project file based on the YAML format [111], which consists of a project name, a model name, a reference to the dataset file, a reference to the label map file, a reference to the attribution files, and a reference to the analysis files. The project and model name can be chosen arbitrarily and are only used to display them in the user interface of *ViRelAy*, to distinguish between multiple loaded projects. The dataset consists of (1) an arbitrary name used for informational purposes, (2) a type that determines if the input dataset is stored as an image directory or an HDF5 database, (4) a path to the input data directory or file, (5) the width and (6) height to which the input images are to be rescaled, (7) the up-sampling and (8) down-sampling approach used for rescaling, and (9) the path to the label map JSON file. The attributions property consists of (1) an attribution method, which is the name of the approach used to compute the attributions, (2) the attribution strategy to indicate whether the true label or the predicted label was attributed, and (3) a list of source files. Finally, the analyses property is a list of analyses that were performed on the data. Multiple analyses can be created to compare different analysis methods. Each analysis consists of the name of the analysis method and a list of source files.

A complete specification of the different HDF5 database formats (https://virelay.rtfd.io/en/1.0.0/contributors-guide/database-specification.html) and the project file format (https://virelay.rtfd.io/en/1.0.0/contributors-guide/project-file-format.html), as well as a guide on how to create a *ViRelAy* project from scratch (https://virelay.rtfd.io/en/1.0.0/user-guide/how-to-create-a-project.html) can be found in our documentation.

### Layer-wise relevance propagation: Details

The most basic LRP propagation rule is the ${\text{LRP}}_{0}$ rule [9] (cf. Eq 1), which redistributes the relevance of a neuron to the neurons in the previous layer based on their activation values and weights.

$$R_{i}^{\left(l-1\right)}=\sum_{j=1}^{n^{\left(l\right)}}\frac{a_{i}w_{ij}}{b_{j}+\sum_{i^{\prime }=1}^{n^{\left(l-1\right)}}a_{i^{\prime }}w_{i^{\prime }j}}R_{j}^{\left(l\right)}\text{,}$$ (1)

$R_{i}^{\left(l-1\right)}$ is the relevance of the $i^{\text{th}}$ neuron in the layer prior to layer *l* onto which part of the relevance of all neurons it is connected to in layer *l* is redistributed. $R_{j}^{\left(l\right)}$ is the relevance of the $j^{\text{th}}$ neuron in layer *l*, which is one of the neurons from which the $i^{\text{th}}$ neuron is receiving relevance. *a*_*i*_ is the activation of the $i^{\text{th}}$ neuron and *w*_*ij*_ is the weight of the connection between the $i^{\text{th}}$ and the $j^{\text{th}}$ neuron. The sum in the denominator normalizes the fraction of relevance the $i^{\text{th}}$ neuron receives from the $j^{\text{th}}$ neuron, in order to satisfy the conservation of relevance. It sums the activations of all neurons $j^{\prime }$ from layer *l* that the $j^{\text{th}}$ neuron is connected with, weighted by weight $w_{i^{\prime }j}$ of their connection. This rule makes a lot of sense, as it redistributes part of the relevance of the neurons in a layer to a neuron in the previous layer based on how it contributed to their activation. Unfortunately, it can be shown that this rule is equivalent to the basic Gradient×Input method that produces poor explanations, which can be attributed to the fact that gradients in DNNs are often noisy [10].

As a first remedy, the ${\text{LRP}}_{\varepsilon }$ rule [9] (cf. Eq 2) was devised, which extends the basic ${\text{LRP}}_{0}$ rule by adding a small positive term ε to the denominator. This helps absorb weak or contradictory explanations, leading to sparser and less noisy results as ε increases [10].

$$R_{i}^{\left(l-1\right)}=\sum_{j=1}^{n^{\left(l\right)}}\frac{a_{i}w_{ij}}{\varepsilon_{\pm }+b_{j}+\sum_{i^{\prime }=1}^{n^{\left(l-1\right)}}a_{i^{\prime }}w_{i^{\prime }j}}R_{j}^{\left(l\right)}\text{,}$$ (2)

where $\varepsilon_{\pm }=\varepsilon \;\cdot \;\text{sign}\;(b_{j}+\sum_{i^{\prime }=1}^{n^{\left(l-1\right)}}a_{i^{\prime }}w_{i^{\prime }j})$. The stabilizer ε, however, introduces the problem, that it absorbs some of the relevance, which means that the conservation property of LRP no longer holds, unless it is reformulated to a weaker version. Bach et al. introduce the ${\text{LRP}}_{\alpha \beta }$ rule [9] (cf. Eq 3), which stabilizes the explanation without leaking relevance. It treats positive and negative relevance contributions separately using two parameters α and β, where α+β=1, which also means that the influence of positive and negative contributions can be manually controlled by choosing suitable values for α and β.

$$R_{i}^{\left(l-1\right)}=\sum_{j=1}^{n^{\left(l\right)}}\left(\alpha \frac{(a_{i}w_{ij}{)}^{+}}{b_{j}^{+}+\sum_{i^{\prime }=1}^{n^{\left(l-1\right)}}(a_{i^{\prime }}w_{i^{\prime }j}{)}^{+}}-\beta \frac{(a_{i}w_{ij}{)}^{-}}{b_{j}^{-}+\sum_{i^{\prime }=1}^{n^{\left(l-1\right)}}(a_{i^{\prime }}w_{i^{\prime }j}{)}^{-}}\right)R_{j}^{\left(l\right)}$$ (3)

A further improvement, introduced by Montavon et al. is the ${\text{LRP}}_{\gamma }$ rule [10,112] (cf. Eq 4), which, instead of striking a balance between positive and negative contributions, favors positive contributions over negative ones by introducing a factor γ. This helps deliver more stable explanations and reduces the influence of negative relevances as γ increases. In the limit as γ→∞, the ${\text{LRP}}_{\gamma }$ rule becomes equivalent to the ${\text{LRP}}_{\alpha \beta }$ rule with α=1 and β=0.

$$R_{i}^{\left(l-1\right)}=\sum_{j=1}^{n^{\left(l\right)}}\frac{a_{i}\left(w_{ij}+\gamma w_{ij}^{+}\right)}{b_{j}+\gamma b_{j}^{+}+\sum_{i^{\prime }=1}^{n^{\left(l-1\right)}}a_{i^{\prime }}\left(w_{i^{\prime }j}+\gamma w_{i^{\prime }j}^{+}\right)}R_{j}^{\left(l\right)}$$ (4)

There are also more specialized propagation rules, e.g. the ${\text{LRP}}_{♭}$ (pronounced “LRP-flat”, ♭ being the symbol used in musical notation for lowering the pitch by a chromatic semitone) rule [12,113] (cf. Eq 5), which distributes the relevance of a neuron uniformly to the neurons in the previous layer, thus effectively “skipping” the layer. The rule has seen application in many different scenarios. For instance, Bach et al. control the resolution and thereby the semantics of the produced heatmaps by choosing a cut-off point from which on the relevance propagation is no longer influenced by the activations or weights of the layers by using the ${\text{LRP}}_{♭}$ rule [113]. Lapuschkin (To avoid confusion, note that Bach is the birth name of Sebastian Lapuschkin. He is referred to both by his current and his birth name for consistency with the names used in the respective original publications.) et al. compare models that have disparate filter sizes in the bottom-most convolutional layers and therefore employ the ${\text{LRP}}_{♭}$ rule to make the granularity of the heatmaps more comparable [114].

$$R_{i}^{\left(l-1\right)}=\sum_{j=1}^{n^{\left(l\right)}}\frac{1}{n^{\left(l\right)}}R_{j}^{\left(l\right)}$$ (5)

When LRP was first conceived, the propagation rules were designed heuristically. It can, however, be interpreted within the framework of DTD, which uses first-order Taylor expansions of each neuron’s function to obtain linear approximations for them in terms of partial derivatives w.r.t. their inputs. For example, the following general propagation rule can be found by performing a first-order Taylor expansion for a ReLU neuron:

$$R_{i}^{\left(l-1\right)}=\sum_{j=1}^{n^{\left(l\right)}}\frac{w_{ij}(a_{i}-{\tilde{a}}_{i})}{\sum_{i^{\prime }=0}^{n^{\left(l-1\right)}}w_{i^{\prime }j}(a_{i^{\prime }}-{\tilde{a}}_{i^{\prime }})}R_{j}^{\left(l\right)}$$ (6)

Please note that the bias was folded in to the weight tensor, where $w_{ji}=b_{j}$ and *a*_*i*_ = 1 for *i* = 0, which is necessary because the bias would otherwise vanish during differentiation. Evaluated at well-chosen reference points $\tilde{x}$, these linear approximations yield different propagation rules. For example, given the first-order Taylor expansion shown in Eq 6, the reference point $\tilde{a}=0$ yields the ${\text{LRP}}_{0}$ rule [10]:


$$R_{i}^{\left(l-1\right)}={\sum_{j=1}^{n^{\left(l\right)}}\frac{w_{ij}(a_{i}-{\tilde{a}}_{i})}{\sum_{i^{\prime }=0}^{n^{\left(l-1\right)}}w_{i^{\prime }j}(a_{i^{\prime }}-{\tilde{a}}_{i^{\prime }})}R_{j}^{\left(l\right)}|}_{\tilde{a}=0}$$

$$=\sum_{j=1}^{n^{\left(l\right)}}\frac{w_{ij}(a_{i}-0)}{\sum_{i^{\prime }=0}^{n^{\left(l-1\right)}}w_{i^{\prime }j}(a_{i^{\prime }}-0)}R_{j}^{\left(l\right)}$$

$$=\sum_{j=1}^{n^{\left(l\right)}}\frac{a_{i}w_{ij}}{\sum_{i^{\prime }=0}^{n^{\left(l-1\right)}}a_{i^{\prime }}w_{i^{\prime }j}}R_{j}^{\left(l\right)}$$

$$\equiv \sum_{j=1}^{n^{\left(l\right)}}\frac{a_{i}w_{ij}}{b_{j}+\sum_{i^{\prime }=1}^{n^{\left(l-1\right)}}a_{i^{\prime }}w_{i^{\prime }j}}R_{j}^{\left(l\right)}$$

Both ${\text{LRP}}_{\varepsilon }$ and ${\text{LRP}}_{\gamma }$ can also be recovered by choosing suitable reference points, while ${\text{LRP}}_{\alpha \beta }$ can only be recovered for the special case of α=1 and β=0. As ${\text{LRP}}_{♭}$ is a special propagation rule for “skipping” layers that entirely disregards both activations and weights, it can unsurprisingly not be recovered using DTD.

Equipped with this new tool, further propagation rules can be devised. For choosing reference points, Montavon et al. consider two general cases: unconstrained and constrained input space. In the case of an unconstrained input space, i.e. ${\mathbb{R}}^{d}$, a reference point that has the smallest Euclidean distance to the data point results in the *w*^2^ propagation rule [43]:

$$R_{i}^{\left(l-1\right)}=\sum_{j=1}^{n^{\left(l\right)}}\frac{w_{ij}^{2}}{\sum_{i^{\prime }=1}^{n^{\left(l-1\right)}}w_{i^{\prime }j}^{2}}R_{j}^{\left(l\right)}$$ (7)

In the case of a restricted input space, there are numerous conceivable sub-cases. Montavon et al. consider two cases: vector spaces restricted to positive real scalars ${\mathbb{R}}_{+}^{d}$ as they occur, for example, after the application of a ReLU, and box-constrained vector spaces, which is a common use case for images with lower and upper bounds for pixel values $ℬ=\left\{\left\{x_{i}\right\}|\forall_{i=0}^{d}\;l_{i}\leq x_{i}\leq h_{i}\right\}$. Restricting the input space means that the reference point with the smallest distance may lie outside of the domain, in which case the search domain must be restricted as well. The restriction to vector spaces with positive real scalars leads to a reference point, which yields the *z*^ + ^ rule [43]:

$$R_{i}^{\left(l-1\right)}=\sum_{j=1}^{n^{\left(l\right)}}\frac{a_{i}w_{ij}^{+}}{\sum_{i^{\prime }=1}^{n^{\left(l-1\right)}}a_{i^{\prime }}w_{i^{\prime }j}^{+}}R_{j}^{\left(l\right)}$$ (8)

The *z*^ + ^ rule is equivalent to the ${\text{LRP}}_{\alpha \beta }$ rule, where α=1 and β=0. Under the box constraint a reference point is chosen that results in the $z^{ℬ}$-rule, which is also known as the box-rule [43]:

$$R_{i}^{\left(l-1\right)}=\sum_{j=1}^{n^{\left(l\right)}}\frac{x_{i}w_{ij}-l_{i}w_{ij}^{+}-h_{i}w_{ij}^{-}}{\sum_{i^{\prime }=1}^{n^{\left(l-1\right)}}x_{i^{\prime }}w_{i^{\prime }j}-l_{i^{\prime }}w_{i^{\prime }j}^{+}-h_{i^{\prime }}w_{i^{\prime }j}^{-}}R_{j}^{\left(l\right)}$$ (9)

Initially, a single LRP rule was uniformly applied to all layers of a NN, which lead to subpar explanations. Since then, it has become best practice to use a *composite strategy*, which means that different LRP propagation rules are applied to layers based on their type or position within the NN. The following list provides a brief overview of some of the contemporary best practices recommended in the literature:

- ${\text{LRP}}_{0}$ – In the *upper layers* NNs generally only have a small number of neurons, which means that the likelihood of them entangling concepts is high. ${\text{LRP}}_{0}$ is close to the function and thus insensitive to these entanglements [10].
- ${\text{LRP}}_{\varepsilon }$ – The ε-rule is mostly used in *fully-connected layers* or *convolutional layers* that are in *middle* or *close to the output* of the NN. The stacking of layers and the sharing of weights can lead to spurious feature attributions, which are filtered out by the ε-rule. Most commonly, the rule is used with ε≪1, e.g. ε=0.01 [10,12].
- ${\text{LRP}}_{\alpha \beta }$ – *Convolutional layers* in the *lower parts* of the NN are often decomposed using the αβ-rule. Common values are either α=1 and β=0, or α=2 and β=−1 [12].
- ${\text{LRP}}_{\gamma }$ – Abstract concepts formed in the *lower layers* of a NN can usually not be attributed to single pixels in the input. For this reason, the γ-rule is most often used for *convolutional layers* that are *close to the output*, because they spread the relevance uniformly to the whole feature, which makes the heatmaps easier to interpret [10].
- ${\text{LRP}}_{♭}$ – There are multiple common scenarios where the ♭-rule is used: (1) for *convolutional layers near the input* of the NN, thus acting as a cut-off point with the intent of controlling the resolution and semantics of the produced heatmaps or to make CNNs with different depths and filter sizes more comparable, or (2) for the *input layer* [12].
- $w^{2}$ – Since the activations are completely ignored, this rule is used for determining the importance of input features in the *first layer* [43].
- $z^{+}$ – The *z*^ + ^ rule is used for *fully-connected* and *convolutional layers* that are positioned in the *upper parts* of the NN, where neuron activations are positive [43].
- $z^{ℬ}$ – The box rule was specifically designed for input domains with a box constraint, therefore it is mostly used in the *first layer* in NNs with images as inputs [43].

### Spectral relevance analysis: Details

In general, the SpRAy pipeline includes the following steps [20,21]:

1. Performing inference on the desired subset of training samples using the model of interest.
2. Computation of the attribution maps using LRP, which are then pre-processed. Pre-processing steps may include, among others, spatially pooling the relevance values to reduce map size and thereby computational complexity, depth pooling to reduce the number channels, padding to unify map sizes, and normalizing.
3. Spectral cluster analysis of the attribution maps using, for example, the Euclidean distance or the Structural Similarity Index (SSIM) [115] as a metric, and a clustering technique, such as *k*-means clustering [116], Density-Based Spatial Clustering of Applications with Noise (DBSCAN) [117], Hierarchical Density-Based Spatial Clustering of Applications with Noise (HDBSCAN) [118], or Agglomerative Hierarchical Clustering (for an overview, refer to [119]).
4. Identification of interesting clusters:
  (a) For a low number of classes by analyzing the eigengaps, i.e. a drastic increase in difference between successive eigenvalues, sorted in ascending order [120].
  (b) For a high number of classes by comparing the linear separability of the clusters using, e.g., Fisher discriminant analysis [121,122].
5. Optionally, the analyzed samples can be embedded using visual embedding methods such as t-SNE [123] or Uniform Manifold Approximation and Projection (UMAP) [124], based on the spectral embedding from the spectral cluster analysis, to produce a two-dimensional representation of the data that is easier to interpret for humans.

Spectral cluster analysis [125] (for an in-depth tutorial on Spectral Clustering, please refer to [126]) is a clustering technique, which is at the heart of SpRAy. Spectral clustering is based on similarity graphs, such as a *k*-nearest neighbor graph G=(V,E) with nodes *V* and edges *E*. The set of nodes *V* are the samples $s_{1},\ldots ,s_{n}$ of a dataset 𝒟 (in the case of SpRAy the attribution maps). The set of edges is defined as


$$E=\left\{\left\{s_{i},s_{j}\right\}\in V^{2}\;|\;s_{i}\;\text{is among}\;k\text{-nearest neighbors of}\;s_{j}\;\text{using metric}\;d:V^{2}\to \mathbb{R}\right\}$$

The graph *G* is then converted to an adjacency matrix *A*.


$$A_{ij}=\left\{ \begin{array}{cc} 1 & \text{if}\;\{s_{j},s_{i}\}\in E\\ 0 & \text{else} \end{array}\right.$$

Based on the adjacency matrix *A*, the Laplacian *L* is computed using the following formula:


$$L=\nabla^{2}A=D-A\text{,}$$

where *D* is the diagonal degree matrix that gives us the degree of connectivity of each data point.

$$\text{deg}(\nu_{i})=\sum_{j}\left\{ \begin{array}{cc} 1 & \text{if}\;\{\nu_{i},\nu_{j}\}\in E\\ 0 & \text{else} \end{array}\right.$$ (10)

$$D_{ij}=\left\{ \begin{array}{cc} \text{deg}(s_{i}) & \text{if}\;i=j\\ 0 & \text{else} \end{array}\right.$$ (11)

The eigendecomposition of the Laplacian *L* yields a set of eigenvectors $\nu_{1},\ldots ,\nu_{n}$ with corresponding eigenvalues $\lambda_{1},\ldots ,\lambda_{n}$, where the number of $\lambda_{i}=0$ represents the number of disjoint clusters within the dataset. In real-world data the clusters are usually at least loosely connected, therefore, the clusters can be identified by eigenvalues close to 0 followed by an eigengap [20]. Any arbitrary clustering algorithm can then be used to assign cluster labels to the analyzed samples based on the eigenvectors.

In SpRAy, spectral clustering is usually performed on a per-class basis. The resulting spectral clusterings and their optional t-SNE embeddings can then be used to identify anomalous prediction strategies or CH behavior of the model. This is done by first inspecting the eigenvalues of the clusterings. Clusterings with eigenvalues close to zero and well-pronounced eigengaps are good candidates for further inspection. Well-separated clusters within a clustering can often times be interpreted as distinct classification strategies learned by the model. If a cluster is also small and dense, it can be an indication that the cluster represents an anomalous prediction strategy or CH behavior, based on spurious correlations in the data, e.g. features that are co-occurring with a class but are not representative of the class. This reduces the analysis to just a few samples that have to be inspected manually, instead of having to analyze an entire dataset by hand.

### Additional Zennit attribution heatmaps

**Fig 13:**
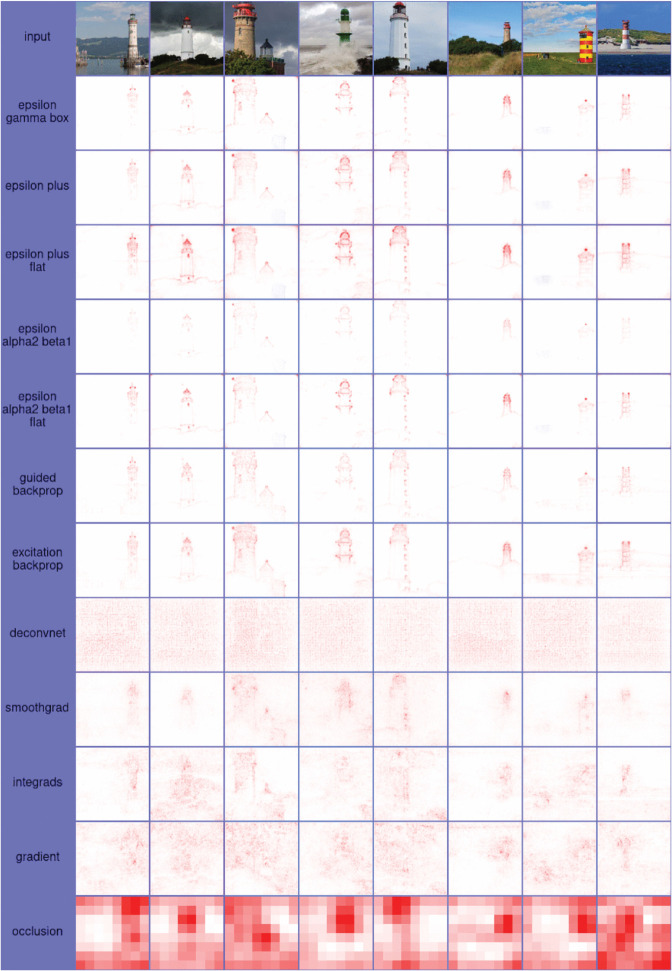
Heatmaps of attributions of lighthouses for VGG16. The attribution scores were computed for the pre-trained VGG-16 network with BatchNorm provided by Torchvision. The model correctly predicted all images as class “lighthouse”. The attributions were visualized with the color map coldnhot (negative relevance is light-/blue, irrelevant pixels are black, positive relevance is red to yellow).

**Fig 14:**
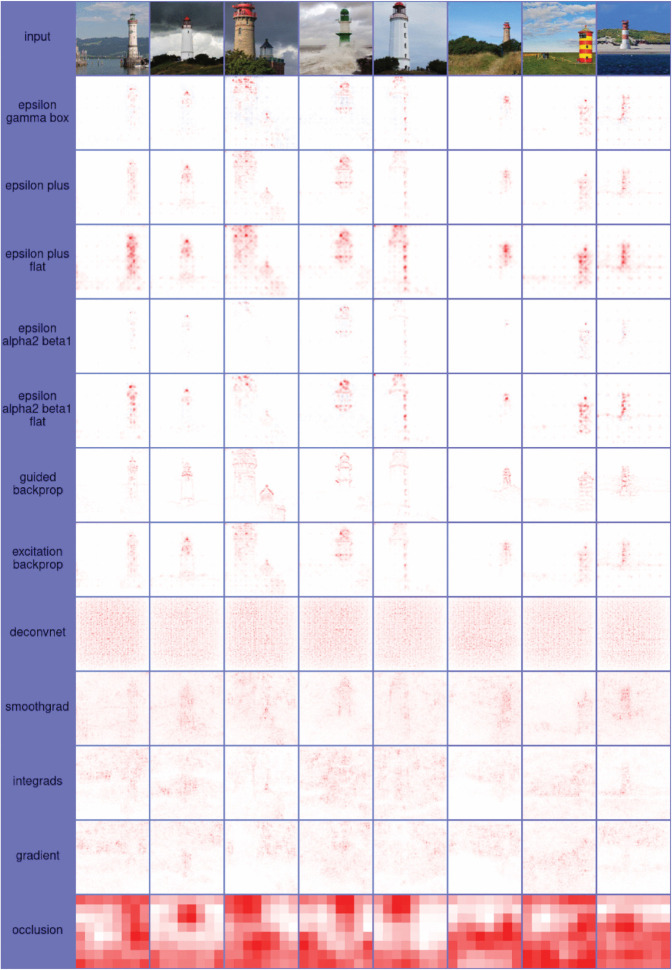
Heatmaps of attributions of lighthouses for ResNet50. The attribution scores were computed for the pre-trained ResNet50 network provided by Torchvision. The model correctly predicted all images as class “lighthouse”. The attributions were visualized with the color map coldnhot (negative relevance is light-/blue, irrelevant pixels are black, positive relevance is red to yellow).

Fig 13 shows attribution heatmaps of the same Torchvision VGG16 model for various methods computed using *Zennit*. Fig 14 shows attribution heatmaps of Torchvision’s ResNet50 model for the same methods also computed using *Zennit*.
