# Supplementary material for: Software for dataset-wide XAI: From local explanations to global insights with Zennit, CoRelAy, and ViRelAy
Source: PLoS One. 2026 Jan 2;21(1):e0336683. doi: 10.1371/journal.pone.0336683 (PMC12758825; doi:10.1371/journal.pone.0336683)
Supplement: Attachment — Submitted filename: Response to Reviewers.pdf [file pone.0336683.s001.pdf]

Software for dataset-wide XAI: From local explanations to global insights with Zennit, CoRelAy, and ViRelAy

Rebuttal Letter: PONE-D-23-05858

Anders, Christopher J. Neumann, David Samek, Wojciech
Müller, Klaus-Robert Lapuschkin, Sebastian

September 30, 2025

Dear Prof. Dr. Galletta,

Please find enclosed our revised manuscript entitled *"Software for dataset-wide XAI: From local explanations to global insights with Zennit, CoRelAy, and ViRelAy"* by C. J. Anders, D. Neumann, W. Samek, K.-R. Müller and S. Lapuschkin (PONE-D-23-05858).

First of all, we would like to express our sincerest apologies for the delay in resubmitting our paper in response to the feedback of the academic editor and the reviewers. Despite our best efforts to address the concerns and suggestions provided, unforeseen circumstances have hindered our progress, including team members completing their PhDs, welcoming new family additions, and relocating to a different continent, which affected our ability to collaborate efficiently. In light of these extraordinary circumstances, we regret that our response has been delayed. We understand the importance of timely communication and the impact it can have on the peer-review process. Please be assured that we have not taken this delay lightly, but we have required the additional time to complete our work without compromising its quality.

We are grateful to the academic editor and reviewers for their thorough reviews and insightful comments, which have undoubtedly helped us improve our manuscript considerably. The points raised were carefully taken into consideration and we believe that the enclosed revision of our manuscript thoroughly addresses them. In the following, we will answer all comments individually. We would like to ask you to kindly reconsider it for publication in PLOS ONE. The revisions made to this updated version of the manuscript are significant enough that they cannot be adequately represented by highlighting additions and deletions. As such, we have chosen not to include a tracked changes document.

In addition to revising the paper, we have also made significant advancements in our software packages. Recent updates and community engagement have led to notable growth, with 283 stars and 14 contributors on GitHub, as well as 88,442 total downloads on PyPI (with 4,579 last month, as reported by `https://clickpy.clickhouse.com/`).

All authors have approved the revision and agree with its resubmission to PLOS ONE.

Yours sincerely,
Prof. Dr. Wojciech Samek,
Prof. Dr. Klaus-Robert Müller,
& Dr. Sebastian Lapuschkin
(on behalf of co-authors).

Reply to the Academic Editor's Comments

Academic Editor Comment 1

Please, consider carefully all the comments of reviewers and implement all the changes they require. In particular, as noted by Reviewer 2 the paper is missing a comprehensive validation of the three tools with well-designed and reproducible experiments on benchmarking tasks. Please, note that addressing this point is crucial for a possible acceptance of the revised version of the manuscript.

We appreciate the editor's guidance and acknowledge the reviewers' concerns. However, we would like to clarify that our paper is primarily focused on presenting software packages for Explainable Artificial Intelligence (XAI), which are being released to the community and the wider public. As such, it's not feasible or necessary to conduct novel experiments to validate these tools. Instead, we've implemented a comprehensive suite of unit tests with full test coverage, static code analysis tools, and a continuous integration pipeline using GitHub actions. We have added a section describing the details of our testing framework.

Additionally, our software packages build upon existing methods that have been peer-reviewed in the literature, *e.g.*, Zeiler and Fergus (2014); Bach et al. (2015); Springenberg et al. (2015); Montavon et al. (2017); Smilkov et al. (2017); Sundararajan et al. (2017); Zhang et al. (2018); Lapuschkin et al. (2019); Kohlbrenner et al. (2020); Pahde et al. (2023a); Motzkus et al. (2022); Anders et al. (2022). We've included examples demonstrating how each package works and have provided extensive documentation. Examples presented in the manuscript are also included in the documentation and therefore fully reproducible.

Our software frameworks have been validated through their extensive use in various experimental setups that are far more involved, *e.g.*, as conducted by Becking et al. (2020); Slijepcevic et al. (2021); Letzgus et al. (2022); Anders et al. (2022); Ede et al. (2022); Achtibat et al. (2023); Weber et al. (2023); Chormai et al. (2024); Hedström et al. (2024); Klauschen et al. (2024); Wagner et al. (2024); Dreyer et al. (2025); Gururaj et al. (2025); Sztukiewicz et al. (2025); Wang et al. (2025); Weber et al. (2025) and others.

Furthermore, the software packages presented in the paper address specific gaps in existing tools, such as complete Layer-wise Relevance Propagation (LRP) implementation for PyTorch with modular composition and canonization, or use-cases for which no prior software packages were available, such as Spectral Relevance Analysis (SpRAy) (Lapuschkin et al., 2019; Anders et al., 2022), a workflow bridging local and global XAI, and *ViRelAy*, a tool for visually inspecting the results of SpRAy pipelines. These tools are designed to be accurate and reliable, as their primary goal is to provide a robust solution for tasks that were previously unaddressed. Benchmarking them against other tools may not be meaningful in this context, as they serve different purposes and address distinct scenarios. While we've made comparisons to other relevant software packages where possible, our primary goal is to make these tools available and known to the scientific community, allowing researchers to easily integrate them as part of their workflows in order to enable the correct and application and representation of the capabilities of methods from XAI in cutting edge science. This approach is consistent with the publication of other widely used scientific software packages.

Nevertheless, we have added a new experiment to Section 7, which verifies our SpRAy implementation on the CIFAR-10 by poisoning the dataset and showing that our implementation produces correct results.

Academic Editor Comment 2

Please ensure that your manuscript meets PLOS ONE's style requirements, including those for file naming.
Please update your submission to use the PLOS LaTeX template.

We have updated the style of the manuscript to the PLOS LaTeX template to ensure compliance.

Academic Editor Comment 3

Please provide an amended statement that declares ***all*** the funding or sources of support (whether external or internal to your organization) received during this study, as detailed online in our guide for authors at `http://journals.plos.org/plosone/s/submit-now`.
Please also include the statement "There was no additional external funding received for this study." in your updated Funding Statement.
Please include your amended Funding Statement within your cover letter. We will change the online submission form on your behalf.

After thorough examination of our Funding Statement, we confirm that all funding sources are accounted for. We have added a slightly updated Funding Statement to the bottom of the Rebuttal Letter (*cf.* Section 3) with the additional statement.

Academic Editor Comment 4

We note that Figure 4 includes an image of a [patient / participant / in the study].
As per the PLOS ONE policy on papers that include identifying, or potentially identifying, information, the individual(s) or parent(s)/guardian(s) must be informed of the terms of the PLOS open-access (CC-BY) license and provide specific permission for publication of these details under the terms of this license. Please download the Consent Form for Publication in a PLOS Journal. The signed consent form should not be submitted with the manuscript, but should be securely filed in the individual's case notes. Please amend the methods section and ethics statement of the manuscript to explicitly state that the patient/participant has provided consent for publication: "The individual in this manuscript has given written informed consent (as outlined in PLOS consent form) to publish these case details".
If you are unable to obtain consent from the subject of the photograph, you will need to remove the figure and any other textual identifying information or case descriptions for this individual.

We have obtained written permission for the publication of these photographs from their author. Please note that due to intensive reworks of parts of our submission the original Figure 4 is now Figure 11.

Reply to the Reviewers' Comments

We appreciate the reviewers' helpful comments and excellent suggestions, which without question have helped us improve our manuscript considerably. We have carefully revised our manuscript accordingly. In the following, please find the detailed answers to the reviewers' remarks and suggestions, and a description of the changes and improvements to be found in the revised manuscript.

1 Reviewer #1's Comments

Reviewer #1 Comment 1

The paper introduces 3 frameworks for Explainable Artificial Intelligence. The first framework provides a highly customisable yet intuitive local XAI framework for PyTorch. It is focused on rule-based approaches such as LRP and based on PyTorch's Module structure, enabling (and delivering) implementations of various attribution methods. The second digests attributions and can be used to quickly elaborate dataset-wide analysis pipelines consisting of, for instance, processing, clustering and embedding steps. The last one provides a user-friendly entry point to the analysis results from the previous two as an interactive web application. The paper discusses their implementation details and highlights some possible analyses of the explainability process in the experimental section.
PRO:
The paper is generally easy to follow and understand; it faces a very hot problem largely discussed in the community, and providing a set of tools for the end-to-end analysis and investigation is powerful and largely convenient. The results highlight the need for this kind of tool to let users understand which are the possible causes of some classification errors. Therefore, they can take actions to remediate.

We thank the reviewer for their positive, as well as constructive feedback, and would like to express our gratitude for the detailed remarks. Points of criticism raised by Reviewer #1 are addressed in detail below.

Reviewer #1 Comment 2

CONS:
In some cases, the authors do not provide useful details that can help the reader understand the proposed solution at its best. For instance, the definition of attribution can be formally provided and possibly add some references (see the sentence in the introduction "Here, attribution methods are able to yield local explanations, *i.e.*, attribution scores for all (input) features of individual samples"). Some details regarding the implementation need to be introduced for readers that might not be familiar with PyTorch. I suggest adding a section where the authors carefully introduce the main concepts used in the rest of the paper.

In order to provide the reader with sufficient information about the background of the XAI methods cited in the paper, we have extended our Related work section with in-depth information about XAI, with a dedicated focus on the feature attribution method LRP, as well as SpRAy, which bridges the gap between local XAI methods such as LRP and global XAI methods. Further, we have added two sections to the appendix that delve deeper into the technical details of LRP and SpRAy. To aid in the understanding of the concepts, we

have added additional technical figures illustrating methods and workflows.

Finally, we have added details about how the automatic differentiation engine of PyTorch works and how we use its `Module` and `Hook` concepts to implement rule-based attribution methods such as LRP in *Zennit*.

Reviewer #1 Comment 3

Adding figures can also help understand the interaction between the PyTorch components.

We agree that some of the concepts are very abstract and are more readily understood when they are visualized. Therefore, in order to aid in the understanding of the concepts, we have added an overview figure to visualize the process of computing feature attributions with *Zennit*.

Reviewer #1 Comment 4

This is also reflected in the related work section, which is quite limited. It introduces similar works, but seeing a larger overview of the state-of-the-art methods might be interesting to understand where this paper can be allocated.

Regarding the related work section, we believe that the focus on literature about related software packages instead of providing a thorough review of XAI methods. Nevertheless, we understand that it is important to provide an overview of the state-of-the-art. For this reason, we have referenced relevant methods in the related work section. Further we have added a new section to the appendix providing an overview of the different methods discussed in the paper. We are confident that the information provided and referenced literature constitutes a thorough introduction of other frameworks and tools that aid researchers and practitioners in the application of XAI methods. For the interested reader, we have referenced multiple review papers, such as Guidotti et al. (2019); Samek et al. (2019); Arrieta et al. (2020); Samek et al. (2021); Holzinger et al. (2022), as well as papers on all methods mentioned in the paper, *e.g.*, Bach et al. (2015); Montavon et al. (2019); Kohlbrenner et al. (2020); Springenberg et al. (2015); Zhang et al. (2018); Lapuschkin et al. (2019); Anders et al. (2022). Along with the new section on the basic concepts of XAI, which should provide the reader a working understanding of all relevant topics that are required to follow the paper, as well as numerous of references for further study.

Reviewer #1 Comment 5

The authors needed to explain more about the differences between their approach and SpRAy. They need to elaborate more on this aspect.

Thank you for your comment. To clarify the scope of our work in relation to SpRAy: this paper focuses on introducing three software packages that, among other functionalities, provide implementations of established methods, including SpRAy (Lapuschkin et al., 2019) and its variants (Lapuschkin et al., 2019). Our intention is not to propose a new variant or method, but rather to offer a comprehensive and accessible software framework that supports the application and integration of such approaches. We have updated the manuscript to better emphasize this distinction and hope this provides greater clarity regarding the contribution of our work. While *Zennit* is a general-purpose XAI framework, *CoRelAy* may be used to

create and execute various data processing pipelines focussed on XAI and *ViRelAy* serving as a platform for visualizing and investigating the other packages' outputs. The explicit goal of the three software packages is to be used in conjunction, which enables users to easily evaluate machine learning models using SpRAy and its variants, and to inspect the results in a graphical user interface to make the detection of Clever Hans (CH) behavior of models as fast and easy as possible. Our implementation is the first general, user-friendly, modular, and customizable implementation of the SpRAy method with a graphical user interface, aiming to support researchers and practitioners in its application.

We have revised our paper accordingly to make our intentions more clear. In addition, we have added a short introduction to XAI in general, and the LRP and SpRAy methods in particular. We now emphasize that these are pre-existing techniques that have already been published in the literature.

Reviewer #1 Comment 6

The experimental section is quite minimal, considering that the paper proposes three frameworks. For instance, it could be interesting to see when some hyper-parameters might affect some results. The provided example shows some classification errors due to some peculiar features of the images. Is it possible to get this outcome with other parameters, or do they need to be tuned? Moreover, Figure 3 could be explained more, carefully describing the meaning of the colours of the dots. Experiments with another dataset could be helpful as well.

We appreciate the reviewer's diligence in thoroughly evaluating our paper. However, we would like to respectfully clarify that our manuscript is not intended to present novel or improved techniques in XAI. Instead, our goal is to provide software packages that enable researchers and practitioners in the field to easily employ these XAI methods. Implementations for these methods were previously either completely unavailable or only available in a limited capacity. This paper is an attempt to contribute to the scientific community's understanding and adoption of these tools, and to provide a comprehensive overview and documentation of their features, capabilities and potential applications.

The primary goal of Section 6, rather than providing novel experimental results, was to reproduce previously published work in order to demonstrate how our frameworks can be employed by the scientific community to easily perform global XAI using SpRAy and to how evaluate and interpret the resulting information. In-depth experiments concerning different variants of LRP, as well as the impact of different hyperparameter configurations may be found in (Samek et al., 2021; Pahde et al., 2023b), which both make use of our software frameworks. Figures 5 and 6 in our paper provide a qualitative overview over some of the implemented feature attribution approaches. SpRAy has also been thoroughly experimentally validated, as can be seen in (Lapuschkin et al., 2019; Anders et al., 2022).

However, to provide further evidence of the correctness of our frameworks, we have added a section thoroughly explaining our testing regime that includes unit tests with 100% code coverage, the use of static code analysis tools, and the constant validation via a Continuous Integration (CI) pipeline. Further, we have expanded Section 7 with a novel benchmarking experiment based on CIFAR-10 that has not been previously published, thus further demonstrating that our implementation of SpRAy produces correct results.

We have expanded the explanation of Figure 3 (now Figure 10).

2 Reviewer #2's Comments

Reviewer #2 Comment 1

This manuscript presents three open-source XAI software tools: *Zennit*, *CoRelAy*, and *ViRelAy*.

We thank the reviewer for their constructive feedback. Points of criticism raised by Reviewer #2 are discussed addressed in detail below.

Reviewer #2 Comment 2

While PLOS ONE welcomes submissions that focus on new methods, software, databases, or tools, these contributions must satisfy three criteria: utility, validation, and availability. Regrettably, a comprehensive validation of the three tools is largely absent. Although there is a brief section dedicated to comparing them with existing tools (Section 5), it fails to establish the superiority of the proposed softwares through objective and measurable assessments. I would recommend that the authors establish a robust evaluation framework that encompasses the validation of all existing tools, including those introduced in this manuscript. For instance, if one of the proposed software tools demonstrates significantly higher efficiency and scalability compared to other competitors, this should be empirically demonstrated through well-designed experiments on benchmarking tasks.

The main reason for creating these three software packages was that no other frameworks are available that fulfill our intended purpose. If the feature set of our frameworks was equivalent to others and only distinguished themselves through their efficiency or scalability, we would agree that comprehensive experimental evidence must be provided to substantiate these claims. This is however not the case: None of the comparable frameworks provide the necessary functionality, thus rendering any attempt to compare them in a quantitative fashion futile, as such a comparison would necessarily be limited to the intersection of the feature sets of all frameworks. This would completely disregard the feature-completeness provided by our frameworks in favor of performance or scalability.

Let us consider *Zennit*: None of the other frameworks discussed in Table 1 of Section 6 in the revised version of our manuscropt support the rule-based application of different attribution methods (*e.g.*, based on layer type or position within the computation graph), nor do they support the canonization of neural network models. *Zennit* implements LRP with a level of feature-richness, modularity, and compatibility which was previously not attainable through any implementation. For example, a comparison between Captum and *Zennit* would be limited to trivial LRP variants, without recognizing that *Zennit* offers many more variants of LRP, that are not only more involved, but are impossible to implement using the current architecture of Captum. To compare *Zennit* to any of its competitors would have to result in a partial or complete re-implementation of these features within the respective frameworks, which we believe to be without the scope of our paper.

Likewise, there are many other frameworks for processing data in a pipeline, similar to *CoRelAy*, yet none of them have built-in support for creating XAI pipelines for SpRAy and its variants. Again, to compare them would require a re-implementation of the entirety of our supported XAI approaches in said other frameworks, which would defeat the purpose of creating a specialized implementation.

Similarly, *ViRelAy* provides a functionality for which no alternative implementations exist, and as such, render any comparison impossible.

Proving that our software packages are more performant or scalable than comparable software packages is not our intended contribution and could only be delivered meaningfully when comparing frameworks of equal capabilities. Instead, we intent to provide to the research community for the first time such frameworks bringing forth comprehensive, correct and well-documented implementations of various XAI methods and tool chains.

We do understand, however, the need to properly showcase that our frameworks are fit for their intended purpose. Therefore, we have created a new benchmark experiment based on CIFAR-10, which can be found in Section 7. We inject CH or shortcut learning features into the dataset and then showcase how we can easily uncover spurious classification strategies learned by the model.

Furthermore, we validate each of our frameworks comprehensively using a set of rigorous unit tests with complete code coverage in order to validate their correctness. These are furthermore automated through continuous integration, and are completely publicly accessible on each project's respective GitHub repository. To clarify this validation process, we have added a paragraph to the manuscript detailing these tests for each of the frameworks.

3 Updated Funding Statement

CJA, WS, and KRM acknowledge support by the German Ministry for Education and Research (BMBF) under grants 01IS14013A-E, 01GQ1115, 01GQ0850, 01IS18025A, 031L0207D, and 01IS18037A. (https://www.bmbf.de). DN, WS, and SL acknowledge funding from the European Union's Horizon Europe research and innovation programme (EU Horizon Europe) as grants TEMA (101093003) (https://tema-project.eu) and ACHILLES (101189689) (https://www.achilles-project.eu). WS further acknowledges support by the German Research Foundation (DFG) as research unit DeSBi [KI-FOR 5363] (459422098) (https://www.dfg.de). K.R.M. was partly supported by the Institute of Information & Communications Technology Planning & Evaluation (IITP) grants funded by the Korea government (MSIT) (No.2019-0-00079, Artificial Intelligence Graduate School Program, Korea University and No. 2022-0-00984, Development of Artificial Intelligence Technology for Personalized Plug-and-Play Explanation and Verification of Explanation) (https://www.msit.go.kr).

The funders had no role in study design, data collection and analysis, decision to publish, or preparation of the manuscript.

There was no additional external funding received for this study.

References

M. D. Zeiler and R. Fergus, "Visualizing and understanding convolutional networks," in *European conference on computer vision.* Springer, 2014, pp. 818–833.

S. Bach, A. Binder, G. Montavon, F. Klauschen, K.-R. Müller, and W. Samek, "On pixel-wise explanations for non-linear classifier decisions by layer-wise relevance propagation," *PLoS ONE*, vol. 10, no. 7, p. e0130140, 2015.

J. T. Springenberg, A. Dosovitskiy, T. Brox, and M. A. Riedmiller, "Striving for simplicity: The all convolutional net," in *Proceedings of the International Conference of Learning Representations (ICLR)*, 2015, pp. 1–14.

G. Montavon, S. Lapuschkin, A. Binder, W. Samek, and K.-R. Müller, "Explaining nonlinear classification decisions with deep taylor decomposition," *Pattern Recognition*, vol. 65, pp. 211–222, 2017.

D. Smilkov, N. Thorat, B. Kim, F. B. Viégas, and M. Wattenberg, "Smoothgrad: removing noise by adding noise," *CoRR*, vol. abs/1706.03825, 2017.

M. Sundararajan, A. Taly, and Q. Yan, "Axiomatic attribution for deep networks," in *Proceedings of the 34th International Conference on Machine Learning, ICML 2017, Sydney, NSW, Australia, 6-11 August 2017*, ser. Proceedings of Machine Learning Research, D. Precup and Y. W. Teh, Eds., vol. 70. PMLR, 2017, pp. 3319–3328.

J. Zhang, S. A. Bargal, Z. Lin, J. Brandt, X. Shen, and S. Sclaroff, "Top-down neural attention by excitation backprop," *International Journal of Computer Vision*, vol. 126, no. 10, pp. 1084–1102, 2018.

S. Lapuschkin, S. Wäldchen, A. Binder, G. Montavon, W. Samek, and K.-R. Müller, "Unmasking clever hans predictors and assessing what machines really learn," *Nature Communications*, vol. 10, p. 1096, 2019.

M. Kohlbrenner, A. Bauer, S. Nakajima, A. Binder, W. Samek, and S. Lapuschkin, "Towards best practice in explaining neural network decisions with lrp," in *Proceedings of the IEEE International Joint Conference on Neural Networks (IJCNN)*, 2020, pp. 1–7.

F. Pahde, G. Ü. Yolcu, A. Binder, W. Samek, and S. Lapuschkin, "Optimizing explanations by network canonization and hyperparameter search," in *Proceedings of the IEEE/CVF Conference on Computer Vision and Pattern Recognition*, 2023, pp. 3819–3828.

F. Motzkus, L. Weber, and S. Lapuschkin, "Measurably stronger explanation reliability via model canonization," in *2022 IEEE International Conference on Image Processing (ICIP)*, IEEE. Bordeaux, France: IEEE, 2022, pp. 516–520.

C. J. Anders, L. Weber, D. Neumann, W. Samek, K.-R. Müller, and S. Lapuschkin, "Finding and removing clever hans: Using explanation methods to debug and improve deep models," *Information Fusion*, vol. 77, pp. 261–295, 2022.

D. Becking, M. Dreyer, W. Samek, K. Müller, and S. Lapuschkin, "Ecq x: explainability-driven quantization for low-bit and sparse dnns," in *International Workshop on Extending Explainable AI Beyond Deep Models and Classifiers.* Springer, 2020, pp. 271–296.

D. Slijepcevic, F. Horst, S. Lapuschkin, B. Horsak, A.-M. Raberger, A. Kranzl, W. Samek, C. Breiteneder, W. I. Schöllhorn, and M. Zeppelzauer, "Explaining machine learning models for clinical gait analysis," *ACM Transactions on Computing for Healthcare (HEALTH)*, vol. 3, no. 2, pp. 1–27, 2021.

S. Letzgus, P. Wagner, J. Lederer, W. Samek, K.-R. Müller, and G. Montavon, "Toward explainable artificial intelligence for regression models: A methodological perspective," *IEEE Signal Processing Magazine*, vol. 39, no. 4, pp. 40–58, 2022.

S. Ede, S. Baghdadlian, L. Weber, A. Nguyen, D. Zanca, W. Samek, and S. Lapuschkin, "Explain to not forget: Defending against catastrophic forgetting with xai," in *Machine Learning and Knowledge Extraction*, A. Holzinger, P. Kieseberg, A. M. Tjoa, and E. Weippl, Eds. Cham: Springer International Publishing, 2022, pp. 1–18.

R. Achtibat, M. Dreyer, I. Eisenbraun, S. Bosse, T. Wiegand, W. Samek, and S. Lapuschkin, "From attribution maps to human-understandable explanations through concept relevance propagation," *Nature Machine Intelligence*, vol. 5, no. 9, pp. 1006–1019, 2023.

L. Weber, S. Lapuschkin, A. Binder, and W. Samek, "Beyond explaining: Opportunities and challenges of xai-based model improvement," *Information Fusion*, vol. 92, pp. 154–176, 2023.

P. Chormai, J. Herrmann, K.-R. Müller, and G. Montavon, "Disentangled explanations of neural network predictions by finding relevant subspaces," *IEEE Trans. Pattern Anal. Mach. Intell.*, 2024.

A. Hedström, L. Weber, S. Lapuschkin, and M. Höhne, "A fresh look at sanity checks for saliency maps," in *Explainable Artificial Intelligence*, L. Longo, S. Lapuschkin, and C. Seifert, Eds. Cham: Springer Nature Switzerland, 2024, pp. 403–420.

F. Klauschen, J. Dippel, P. Keyl, P. Jurmeister, M. Bockmayr, A. Mock, O. Buchstab, M. Alber, L. Ruff, G. Montavon *et al.*, "Toward explainable artificial intelligence for precision pathology," *Annual Review of Pathology: Mechanisms of Disease*, vol. 19, no. 1, pp. 541–570, 2024.

P. Wagner, T. Mehari, W. Haverkamp, and N. Strodthoff, "Explaining deep learning for ecg analysis: Building blocks for auditing and knowledge discovery," *Computers in Biology and Medicine*, vol. 176, p. 108525, 2024. [Online]. Available: https://www.sciencedirect.com/science/article/pii/S0010482524006097

M. Dreyer, J. Berend, T. Labarta, J. Vielhaben, T. Wiegand, S. Lapuschkin, and W. Samek, "Mechanistic understanding and validation of large ai models with semanticlens," 2025. [Online]. Available: https://arxiv.org/abs/2501.05398

S. Gururaj, L. Grüne, W. Samek, S. Lapuschkin, and L. Weber, "Relevance-driven input dropout: an explanation-guided regularization technique," 2025. [Online]. Available: https://arxiv.org/abs/2505.21595

L. Sztukiewicz, I. Stępka, M. Wiliński, and J. Stefanowski, "Investigating the relationship between debiasing and artifact removal using saliency maps," 2025. [Online]. Available: https://arxiv.org/abs/2503.00234

J. Wang, R. Zhang, and Q. Li, "Tf-lime : Interpretation method for time-series models based on time–frequency features," *Sensors*, vol. 25, no. 9, 2025. [Online]. Available: https://www.mdpi.com/1424-8220/25/9/2845

L. Weber, J. Berend, M. Weckbecker, A. Binder, T. Wiegand, W. Samek, and S. Lapuschkin, "Efficient and flexible neural network training through layer-wise feedback propagation," *Transactions on Machine Learning Research*, 2025. [Online]. Available: https://openreview.net/forum?id=9oToxYVOSW

R. Guidotti, A. Monreale, S. Ruggieri, F. Turini, F. Giannotti, and D. Pedreschi, "A survey of methods for explaining black box models," *ACM Computing Surveys*, vol. 51, no. 5, pp. 93:1–93:42, 2019.

W. Samek, G. Montavon, A. Vedaldi, L. K. Hansen, and K. Müller, Eds., *Explainable AI: Interpreting, Explaining and Visualizing Deep Learning*, ser. Lecture Notes in Computer Science. Springer, 2019, vol. 11700.

A. B. Arrieta, N. D. Rodríguez, J. D. Ser, A. Bennetot, S. Tabik, A. Barbado, S. García, S. Gil-Lopez, D. Molina, R. Benjamins, R. Chatila, and F. Herrera, "Explainable artificial intelligence (XAI): concepts, taxonomies, opportunities and challenges toward responsible AI," *Inf. Fusion*, vol. 58, pp. 82–115, 2020.

W. Samek, G. Montavon, S. Lapuschkin, C. J. Anders, and K.-R. Müller, "Explaining deep neural networks and beyond: A review of methods and applications," *Proceedings of the IEEE*, vol. 109, no. 3, pp. 247–278, 2021.

A. Holzinger, A. Saranti, C. Molnar, P. Biece, and W. Samek, "Explainable ai methods - a brief overview," in *xxAI - Beyond Explainable AI*, ser. Lecture Notes in Artificial Intelligence, A. Holzinger, R. Goebel, R. Fong, T. Moon, K.-R. Müller, and W. Samek, Eds. Springer International Publishing, 2022, vol. 13200, pp. 13–38.

G. Montavon, A. Binder, S. Lapuschkin, W. Samek, and K.-R. Müller, "Layer-wise relevance propagation: An overview," in *Explainable AI: Interpreting, Explaining and Visualizing Deep Learning*. Springer LNCS 11700, 2019, pp. 193–209.

F. Pahde, G. ümit Yolcu, A. Binder, W. Samek, and S. Lapuschkin, "Optimizing explanations by network canonization and hyperparameter search," in *Proceedings of the IEEE/CVF Conference on Computer Vision and Pattern Recognition Workshops (CVPRW)*, 2023, pp. 3818–3827.
